# Transabdominal photobiomodulation applications: A systematic review and meta‐analysis

**DOI:** 10.1111/obr.13921

**Published:** 2025-04-04

**Authors:** Ana M. Jiménez‐García, Candela Zorzo, Alba Gutiérrez‐Menéndez, Jorge L. Arias, Natalia Arias

**Affiliations:** ^1^ BRABE Group, Department of Psychology, Faculty of Life and Natural Sciences University of Nebrija Madrid Spain; ^2^ Neuroscience Laboratory, Department of Psychology University of Oviedo Oviedo Spain; ^3^ INEUROPA, Instituto de Neurociencias del Principado de Asturias Oviedo Spain; ^4^ ISPA, Instituto de Investigación Sanitaria del Principado de Asturias Oviedo Spain

**Keywords:** abdominal, insulin, obesity, photobiomodulation, triglycerides

## Abstract

**Introduction:**

The escalating prevalence of obesity presents a multifaceted challenge involving genetic, environmental, and behavioral factors, with significant public health implications. Photobiomodulation (PBM) may positively influence metabolic activities in adipose cells and regulate inflammation, potentially impacting obesity.

**Methods:**

A systematic review and meta‐analysis were conducted to assess the effects of transabdominal PBM treatments in preclinical and clinical obesity studies, covering a range of physical, psychological, and physiological variables. Research articles were sourced from PubMed, Web of Science, ScienceDirect, and Scopus databases. Following the inclusion and exclusion criteria, a total of 24 studies, comprising 1041 patients, and 100 mice were incorporated. R software was employed for conducting meta‐analyses, and calculating effect sizes between experimental and control groups.

**Results:**

In human models, significant discrepancies were revealed in waist circumference (Z = ‐2.16; p = 0.031), hip circumference (Z = 2.11; p = 0.035), insulin levels (Z = 2.11; p = 0.035), and triglycerides (Z = ‐2.4674, p = 0.0136). In animal models, significant differences were observed in epididymal adipocyte area (Z = ‐5.6930; p < 0.0001), triglycerides (Z = ‐2.0254; p = 0.04848), and glucose area under the curve (AUC; Z = ‐6.4112; p < 0.0001).

**Conclusions:**

This study underscores the necessity of considering diverse wavelengths in PBM research, particularly within the realm of obesity, and emphasizes the imperative for further investigations to comprehensively elucidate PBM mechanisms and applications. The exploration of innovative therapeutic approaches unfolds novel avenues in the pursuit of comprehensive strategies to address obesity and its underlying determinants.

## 1 INTRODUCTION

Obesity, traditionally defined as excessive fat deposits causing harm to health, stands as one of the most significant global health challenges.^1^ In accordance with data provided by the World Health Organisation (WHO), it is estimated that in 2022 worldwide, approximately 16% of adults aged 18 years and older were living with obesity. This prevalence signifies a more than twofold increase over the preceding 30 years.^2^ Obesity is a complex condition influenced by a combination of factors, including obesogenic environments, psychosocial factors, and genetic predisposition,^3^, ^4^ and is accompanied by an increased risk and earlier onset of certain diseases, such as cardiovascular alterations, hypertension, or type 2 diabetes.^1^

Lifestyle modification is often recommended as the primary approach for managing obesity and may be supplemented with pharmacological treatments or bariatric surgery, depending on the severity of the condition.^5^, ^6^ However, many patients struggle to achieve long‐term benefits due to difficulties with adherence and physiological and hormonal adaptations following weight loss.^5^ Unfortunately, adherence to obesity treatments tends to be low, with high rates of relapse frequently observed.^7^

An alternative approach involves integrating multiple treatment modalities with diverse mechanisms of action, which holds considerable promise for achieving substantial and lasting changes in the body. Recent studies suggest that incorporating light‐based therapies could enhance non‐invasive obesity treatments.^7^, ^8^ Photobiomodulation (PBM) involves the non‐invasive application of photons from an external near‐infrared light source to any anatomical surface. PBM has been used to stimulate, heal, regenerate, and protect damaged tissue,^9^ with recent applications extending to brain,^10^ and abdominal stimulation.^11^ However, the effects of PBM are not limited to these outcomes. Emerging evidence indicates that PBM can modulate the metabolism of adipose tissue and regulate inflammation,^8^, ^11^ offering potential benefits in obesity management. Furthermore, PBM has been shown to reduce postprandial glucose spikes by modulating glucose regulation.^12^

PBM‐induced cellular stimulation promotes lipolysis and apoptosis in superficial adipose tissue, possibly by increasing cyclic adenosine monophosphate (cAMP) levels through the activation of cytochrome c oxidase (CCO), leading to lipid breakdown in adipocytes^13^ and the formation of transient pores in their membranes, ultimately causing cell collapse.^14^ Clinically, light therapy has been used as an adjunct to liposuction, demonstrating its efficacy in reducing operating room time, increasing fat extraction volume, reducing physician effort, and improving patient recovery.^15^ This cascade of events suggests that PBM has a multifaceted effect on mitochondrial and cellular processes related to energy metabolism, and glucose and lipid regulation. Given the central role of disrupted metabolism and glucose homeostasis in obesity and metabolic disorders, PBM presents itself as a potential therapeutic intervention. Moreover, studies indicate that PBM can elicit therapeutic effects without apparent adverse outcomes.^16^ A reduction has been observed in pro‐inflammatory mediators and an increase in anti‐inflammatory mediators with PBM therapy, demonstrating its potential benefits without causing adverse effects.^17^ Chronic inflammation is often observed in obesity, correlating with the development of metabolic diseases and complications in affected individuals. This complexity stems from the intricate interplay of various pro‐ and anti‐inflammatory signaling cascades within the immune response of expanding adipose depots, leading to dysfunctional adipocytes and resulting in adipose tissue inflammation.^18^

However, the effects of PBM depend on the parameters selected. They are highly variable between studies,^19^, ^20^, ^21^ and there is not a consensus about which parameters are optimal for each situation.^20^ Conditions related to wavelengths can significantly influence the therapeutic outcomes of PBM. Various wavelengths exhibit distinct interactions with biological tissues and their components, leading to diverse effects on cellular metabolism, the function of endogenous photoreceptors, and intracellular signaling pathways.^22^ For instance, shorter wavelengths within the near‐infrared (NIR) spectrum, approximately 700–900 nm, are more prone to superficial absorption in the skin. This characteristic makes them well‐suited for applications targeting the stimulation of blood circulation in the epidermis and superficial dermis.^23^ Alternatively, wavelengths in the blue and green light spectrum,^24^ such as approximately 415 and 540 nm, have the ability to stimulate opsins like melanopsin (opsin 4) present in adipocytes, as well as a tri‐stable switch that absorbs in the red spectrum.^25^ This interaction may lead to a distinct modulation of cellular activity compared to neighboring wavelengths.^26^, ^27^

Given the diverse impact of light based on its application site and the specific wavelengths employed, this study endeavored to investigate the potential systemic alterations that can arise from the transabdominal application of PBM in obesity. This exploration sought to unveil anthropometrical, psychological, and physiological variables. Adopting this comprehensive perspective may offer valuable insights into the fundamental mechanisms of PBM and its overall impact on health and well‐being.

## 2 MATERIAL AND METHODS

### 2.1 Research strategy

A systematic literature search was conducted in accordance with the Preferred Reporting Items for Systematic Reviews and Meta‐Analyses (PRISMA) guidelines. Original research articles pertaining to transabdominal PBM applications in obesity were independently searched for in four electronic databases – PubMed, Web of Science (WoS), Science Direct, and Scopus. The search was performed by two researchers (CZ, AGM) on November 22nd, 2023, using the following search terms and combinations: (“photobiomodulation” OR “low level laser therapy” OR “low light laser therapy” OR “low power light therapy” OR “low power laser therapy” OR “laser biostimulation”) AND (“obesity”) NOT (“review”). No chronological nor methodological filters were imposed on the search engines besides filtration of titles, keywords, and abstracts, and all resulting data sets were exported and compiled in Mendeley and Excel.

### 2.2 Study selection

Following the removal of duplicates, all remaining articles had their titles and abstracts screened for eligibility (CZ, AGM). In cases where conflicts arose during the screening process, a consensus was reached through discussion among all the authors involved in the article. Epidemiological studies and articles that did not specifically address transabdominal photobiomodulation in obesity therapies were considered ineligible. After the initial screening phase, the full texts of selected studies were retrieved and reviewed in detail against the inclusion criteria. In order for a study to be included in the systematic review, it had to (i) address the transabdominal application of PBM, and (ii) show clear evidence of changes in any parameters related to its application in preclinical or clinical studies concerning obesity. Specifically, the inclusion and exclusion criteria were defined using the PICOS framework (Population, Intervention, Comparison, Outcomes, and Study Design). The study population comprised individuals with overweight or obesity, as well as animal models with diet‐induced obesity. The intervention involved the transabdominal application of PBM across various wavelengths. Comparisons varied between studies and included control groups with no intervention, placebo treatments, or non‐obese groups. The outcomes measured encompassed anthropometrical changes such as body mass index (BMI), weight, waist, neck and hip circumference, waist‐to‐hip ratio, skinfold thickness, lean mass, and visceral fat. Psychological outcomes included quality of life and body image, while physiological outcomes assessed changes in fasting glucose, lipids, total cholesterol, insulin, Homeostatic Model Assessment for Insulin Resistance (HOMA‐IR), leptin, and platelets. All variables were manually extracted from each study (AJG and NA) and compiled for subsequent meta‐analysis. The study designs incorporated in this review included randomized clinical trials, non‐randomized trials, between‐group experimental designs, and animal studies.

### 2.3 Methods quality

The methodological quality of the clinical research studies included in this review was evaluated using the Cochrane Risk of Bias Assessment Tool.^28^ This structured approach assesses the risk of bias across seven critical domains: participant selection, the randomization process, allocation concealment, blinding of participants and study personnel, blinding of outcome assessors, management of incomplete data, and selective reporting. Each domain was rated as having a low, unclear, or high risk of bias, allowing for a comprehensive evaluation of the methodological integrity of each study. By applying this tool, a robust assessment of the internal validity and reliability of the study outcomes was achieved, ensuring a solid foundation for interpreting the results.

### 2.4 Meta‐analysis

A continuous random effects model with a standard mean difference was employed to conduct the meta‐analysis. Human studies that reported (i) anthropometrical changes, including BMI, weight, waist, neck, and hip circumference, waist‐to‐hip ratio, skinfold thickness, lean mass, and visceral fat; (ii) psychological variables such as quality of life and body image; and (iii) physiological changes, including fasting glucose, lipid levels, total cholesterol, insulin, HOMA‐IR, leptin, and platelet counts were included in this review. Additionally, animal studies that reported body mass, epididymal, mesenteric, or retroperitoneal adipocyte area, cholesterol, triglycerides, insulin, glucose concentration, and HOMA‐IR were also analyzed in the current study. Significance played no role in the selection process, with studies reporting null findings being included by the authors of this review. The authors of the relevant publications were not contacted directly regarding the raw data sets. Instead, numerical data was extracted directly from the figures using the online data extraction tool PlotDigitizer. Means, standard deviations, and sample sizes were entered into R Studio software version 4.3.1.^29^ which automatically calculated the standard mean difference (SMD), confidence intervals (CIs), heterogeneity, and overall effect size using a random effects model. In the heterogeneity analysis of the meta‐analysis, several methods were employed to assess the variability among studies. Cochran's Q statistic and the I^2^ index were utilized to determine the presence of significant heterogeneity. The Egger test was performed when there were more than two studies, while the Begg and Mazumdar's rank correlation test was applied consistently across all analyses to evaluate potential publication bias. Additionally, a funnel plot was generated for analyses that included more than 10 studies, providing a visual representation of symmetry and enabling the detection of potential biases in the literature.

Finally, the code to calculate the Fail‐Safe N based on the Rosenthal approach with the ‘metafor’ package was used to analyze the robustness of the results. For those cases where high variability was found in the data, further sub‐analyses were performed considering the wavelength applied during the intervention. Forest plots were generated using Review Manager (RevMan) software.

### 2.5 Results

**FIGURE 1:**
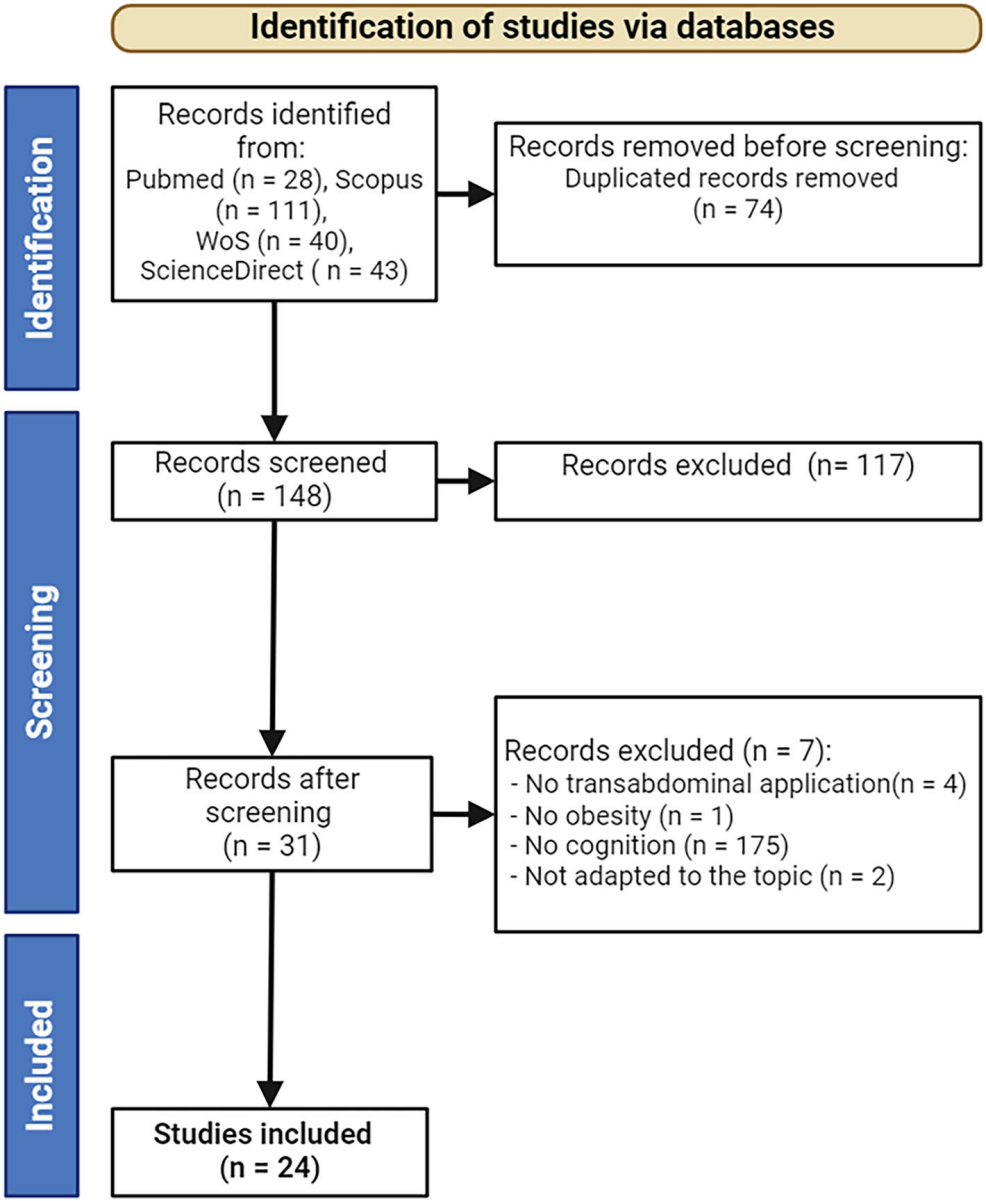
PRISMA flow chart of selection of publications for inclusion in the review. General characteristics of selected studies.

The searches conducted in PubMed, Scopus, Science Direct, and WoS electronic databases yielded 28, 111, 43, and 40 articles, respectively, reaching a total of 222 publications, of which 74 were identified as duplicates and removed from the data set. The titles and abstracts of the remaining 148 articles were screened for eligibility, with 117 publications deemed to fall outside the scope of the systematic review and excluded. Full texts of the final 31 articles were retrieved, read in full, and carefully assessed against the inclusion criteria, with 24 studies deemed eligible for inclusion in the systematic review (see Figure 1). Out of the selected full‐text articles (n = 31), seven were excluded for various reasons, including not assessing the impact of PBM on the abdominal area (n = 4), lack of relevance to the subject of interest (n = 2), and not evaluating the population with obesity (n = 1).

The studies selected for inclusion in the systematic review were published between 2011 and 2023 (n = 24). This study includes 11 randomized clinical trials, 2 experimental between‐group designs, 2 non‐randomized clinical trials, 1 randomized longitudinal prospective study, 1 pre‐test post‐test control group design, and 1 clinical report, most of them on human models (n = 18). Six studies were conducted on animal models, with 1 of them on male Wistar rats, 3 of them on Swiss mice, and 2 of them on male C57BL/6 mice, totaling 100 animals. Within the studies, a total of 1041 patients were evaluated, including people with and without obesity psychopathologies, with a mean age of 36.9 years and a woman/man ratio of 4.91. Most of the studies included samples of between 20 and 157 participants. The remaining studies were single case studies or had a reduced sample size of 5 to 12 participants.

Specifically, human studies included the effectivity of PBM as a treatment on body fat (Croghan et al, 2016^7^; 2020^30^; Elnaggar, 2020^31^; Elsherbeni et al, 2018^32^; Modena et al, 2022^33^; Nagy et al, 2018^34^; 2021^35^; Roche et al, 2017^36^; Tseng et al, 2015^37^), inflammation (da Silveira Campos et al, 2015^38^; 2018^8^; Duarte et al, 2015^39^; Mostafa et al, 2016^40^; Nagy et al, 2021^35^; Silva et al, 2019^41^ for mice), or lipid profile (Hassan et al, 2014^29^; Modena et al, 2023^13^; Said and Elnhas, 2016^42^; Silva et al, 2018^43^ for mice)T (n = 13), being combined with other therapies such as aerobic plus resistance training (n = 3) (da Silveira Campos et al, 2015^38^; 2018^8^; Duarte et al, 2015^39^) or together with abdominal exercises (n = 1) (Elkablawy et al, 2016)^14^ diet (n = 1) (Elm et al, 2011),^44^ cryolipolysis (n = 1) (Mostafa et al, 2016),^40^ or Artificial Neural Network (ANN) (n = 1) (Nagy et al, 2018).^34^ The following studies focused on PBM as a treatment after a gastric bypass (n = 1) (Elkablawy et al, 2016),^14^ liposuction (n = 1) (Roche et al, 2017)^36^ or in patients diagnosed with non‐alcoholic fatty liver disease (NAFLD) (n = 1) (Nagy et al, 2021).^35^ One of them explored surrogates of mitochondrial function in skeletal muscle in mice after PBM therapy (n = 1) (Silva et al, 2019).^41^

Regarding animal studies, PBM was used in high‐fat diet (HFD)‐induced mice to explore the potential mechanisms of PBM in obesity (n = 1) (Guo et al, 2020),^45^ and also, diet‐induced obese rats without diabetes to explore the impact of PBM on biochemical measurements such as total cholesterol, triglycerides, and very low‐density lipoprotein (VLDL) (n = 1) (Paolillo et al, 2021).^46^ Silva et al (2023)^47^ evaluated the effects of PBM on the expression of thermogenesis and lipogenesis‐associated markers in adipose tissue and metabolic parameters of obese mice (n = 1). Finally, Yoshimura et al (2016)^11^ explored the effect of PBM on managing the chronic inflammatory component of obesity and hyperglycemia in an obese animal model.

Some studies include Grade II or III obesity participants, with a BMI ≥ 40 kg/m^2^ or ≥ 35 kg/m^2^ (Modena et al, 2022^33^; 2023^13^) (n = 2). Others select a BMI ≥ 30 kg/m^2^ (Duarte et al, 2015^39^; Hassan et al, 2014^29^; Da Silveria et al, 2015^38^; 2018^8^; Said et al, 2016^42^; Roche et al, 2017^36^; Elsherbeni et al, 2018^32^) (n = 7), and others include a broader perspective by incorporating the concept of overweight as well as obesity (n = 3): Croghan et al (2016)^7^ considers individuals with a BMI greater than 26.9 and less than 40 kg/m^2^, while Croghan et al (2020)^30^ and Tseng et al (2015)^37^ include those with a BMI between 25.0 and 29.9 kg/m^2^. Also, some studies include subjects with a BMI ≤ 30 kg/m^2^ (Elm et al, 2011^44^; Mostafa et al, 2016^40^), but they account for additional factors such as metabolic syndrome (n = 2). Similarly, others include both overweight and obese participants with a BMI range of 25 to 35 kg/m^2^ (Nagy et al, 2018)^34^ (n = 1) or 30 and 35 kg/m^2^ (Nagy et al, 2021)^35^ (n = 1), incorporating additional metabolic indicators like high triglyceride and LDL levels, as well as an elevated waist circumference. Lastly, Elnaggar et al (2020)^31^ categorize sedentary individuals as obese using the BMI > 95th percentile (n = 1), adjusted for age and gender, rather than using a fixed cutoff. It is important to note that most of these variations depend on the classification of obesity based on ethnic or regional standards. Finally, one study does not specify the exact BMI but refers to abdominal obesity (Elkablawy et al, 2016)^14^ (n = 1).

Regarding animal studies, all involve inducing obesity through dietary interventions. Most of them (n = 5) use high‐fat diets, with up to 60% of calories derived from fat to simulate obesity and hyperglycemia in mice and rats (Guo et al, 2020^45^; Paolillo et al, 2021^46^; Silva et al, 2018,^43^ 2019^41^; Yoshimura et al, 2016^11^), and one of them examines obesity in mice using a hyperglycemic diet high in carbohydrates, consisting of condensed milk, refined sugar, and standard diet (Silva et al, 2023).^47^

### 2.6 Photobiomodulation parameters

The majority of the included studies (n = 19) utilized laser light devices with wavelengths predominantly from the red spectrum (660 nm, n = 1; 650 nm, n = 1; 635 nm, n = 5), followed by the NIR spectrum (924 nm, n = 1; 810 nm, n = 1; 808 nm, n = 6; 780 nm, n = 1) and the green light spectrum (532 nm; n = 3). The remaining 5 articles used an LED (light‐emitting diode) device with wavelengths from the red spectrum (660 nm, n = 1 and 630 nm, n = 1), wavelengths of 843 nm NIR (n = 1), and a combination of 850 nm NIR and 630 nm red light (n = 2).

Concerning other PBM parameters, there is considerable variability among studies. Some studies reported optical power with values of 3.15 W (n = 2), 2.7 W (n = 2), 1.44 W (n = 1), 0.508 W (n = 1), 0.30 W (n = 1), 0.15 W (n = 3), 0.1 W (n = 3), 0.099 W (n = 1), 0.085 W (n = 1), 0.08 W (n = 1), 0.06 W (n = 2), 0.017 W (n = 4), 0.01 W (n = 1) and 0.005 W (n = 1) while the remaining two articles did not provide these parameters. Some articles described irradiance or power density of 4.706 W/cm^2^, n = 1; 0.779 W/cm^2^, n = 1; 0.417 W/cm^2^, n = 1; 0.25 W/cm^2^, n = 1; 0.072 W/cm^2^, n = 1; 0.071 W/cm^2^, n = 1; 0.048 W/cm^2^, n = 1; 0.039 W/cm^2^, n = 2; 0.034 W/cm^2^, n = 2; 0.019 W/cm^2^, n = 1; 0.016 W/cm^2^, n = 2; 0.00625 W/cm^2^, n = 3; 0.0034 W/cm^2^, n = 1; 0.00003 W/cm^2^, n = 1, while the remaining seven articles did not provide this parameter. The energy (J) supplied by the devices was reported in 16 articles, and other studies opted for adding fluency or energy density (J/cm^2^) (n = 19).

All the studies applied PBM to the abdominal area, with some also including radiation of the quadriceps, gluteus, and biceps femoral (n = 3), quadriceps and shoulders (n = 1), quadriceps and upper hind limbs (n = 1), thighs (n = 1), waist (n = 1), back (n = 1), and specific acupuncture points according to the theory of traditional Chinese medicine for obesity (n = 2).

Finally, there is considerable diversity in the irradiation time, treatment interval, and duration. The most common time for applying PBM was 30 minutes (n = 6), followed by 16 minutes (n = 3), 20 minutes (n = 2), and 1 hour (n = 2). Treatment durations varied, including 3 months (n = 1), 2 months (n = 1), 16 weeks (n = 3), 12 weeks (n = 4), 8 weeks (n = 2), 6 weeks (n = 2), 4 weeks (n = 3), 2 weeks (n = 3), 20 sessions (n = 1), 7 sessions (n = 2), and 6 sessions (n = 2). A detailed description of all these parameters is provided in Table 1.

**TABLE 1:** Description of PBM parameters found in the described peer‐reviewed studies for each device.

Source	Population/sample	Light‐emitting device	Wavelength (nm)	Optical power (W)	Irradiance (W/cm^2^)	Energy (J)	Fluence (J/cm^2^)	Area	Treatment period
Croghan et al (2016)^7^	Human	Laser	532	0.017	0.0016	91.8	0.177	Upper abdomen	One hour Once/week 12 weeks
Croghan et al (2020)^30^	Human	Laser	532	0.017	0.0016	91.8	0.177	Upper abdomen	One hour Once/week 12 weeks
Da Silveira et al. (2015)^38^	Human	Laser	808	0.1	0.00625	96	6	Abdomen, quadriceps, gluteus, and biceps femoral	16 minutes Twice/week 16 weeks
Da Silveira et al (2018)^8^	Human	Laser	808	0.1	0.00625	96	6	Abdomen, quadriceps, gluteus, and biceps femoral	16 minutes Twice/week 16 weeks
Duarte et al (2015)^39^	Human	Laser	808	0.1	0.00625	96	6	Abdomen, quadriceps, gluteus, and biceps femoral	16 minutes Twice/week 16 weeks
Elkablawy et al (2016)^14^	Human	Laser	635	0.15	‐	‐	‐	Abdomen	30 minutes Twice/week Three months
Elm et al. (2011)^44^	Human	Laser	635	0.06	‐	‐	6.60	Abdomen and thighs	40 minutes Twice/week Two weeks
Elnaggar et al (2020)^31^	Human	Laser	635	0.085	0.0034	1.224	4.08	Abdomen	20 minutes Twice/week Two weeks
Elsherbeni et al. (2018)^32^	Human	Laser	808	0.15	‐	‐	‐	Abdomen	20 minutes Twice/week Two weeks
Guo et al (2020)^45^	Animal (C57BL/6 mice)	Laser	635	0,508	0.072	56.48	8	Abdomen	Two hours Once/day Eight weeks
Hassan et al. (2014)^29^	Human	Laser Acupuncture	808	0.099	‐	‐	‐	Acupuncture points according to the theory of traditional Chinese medicine for obesity	30 seconds Once/week 12 weeks
Modena et al (2022)^33^	Human	LED	630,850	2.7 3.15	0.034 0.039	2.72 3.12	6 4	Left side abdomen	Twice/week Seven sessions
Modena et al. (2023)^13^	Human	LED	630,850	2.7 3.15	0.034 0.039	2.398292.5	0.0397500	Left side abdomen	Twice/week Seven sessions
Mostafa et al (2016)^40^	Human	Laser	924	0.017	‐	‐	3.94	Abdomen	30 minutes Twice/week Two months
Nagy et al (2018)^34^	Human	Laser	635	‐	‐	‐	‐	Abdomen	30 minutes Twice/week Six weeks
Nagy et al (2021)^35^	Human	Laser	660	0.005	0.071	2.600	127.8	Abdomen and waist	30 minutes Twice/week 12 weeks
Paolillo et al (2021)^46^	Animal (Wistar rat)	Laser	808	0.08	4.706	28.8	1.694	Abdomen	Six minutes Twice/week Four weeks
Roche et al. (2017)^36^	Human	Laser	532	0.017	0.00003	4.32	0.36	Abdomen and back	30 minutes Twice/week Four weeks
Said et al. (2016)^42^	Human	Laser	650	1.44	‐	‐	‐	Abdomen	30 minutes Twice/week Six weeks
Silva et al. (2018)^43^	Animal (Swiss albino mice)	Laser	780	0.01	0.25	0.4	10	Quadriceps femoris muscles, upper hind limbs, and abdomen	40 seconds Five days/week 20 sessions
Silva et al. (2019)^41^	Animal (Swiss albino mice)	LED	630	0.30	0.779	12	31.19	Quadriceps muscle, shoulders, and abdomen	40 seconds Five days/week Four weeks
Silva et al. (2023)^47^	Animal (Swiss mice)	LED	660	‐	0.048	‐	5.77	Abdomen	120 seconds Six sessions
Tseng et al (2015)^37^	Human	Laser acupuncture	810	0.15	0.417	1.44	4	Acupuncture points according to the theory of traditional Chinese medicine for obesity	10 seconds/each point Twice/week Eight weeks
Yoshimura et al. (2016)^11^	Animal (C57BL/6 mice)	LED	843	0.06	0.019	18	5.7	Abdomen	300 seconds Six sessions

## 3 RESULTS FROM META‐ANALYSIS

### 3.1 Human models

#### 3.1.1 Anthropometric variables

##### Weight

**FIGURE 2:**
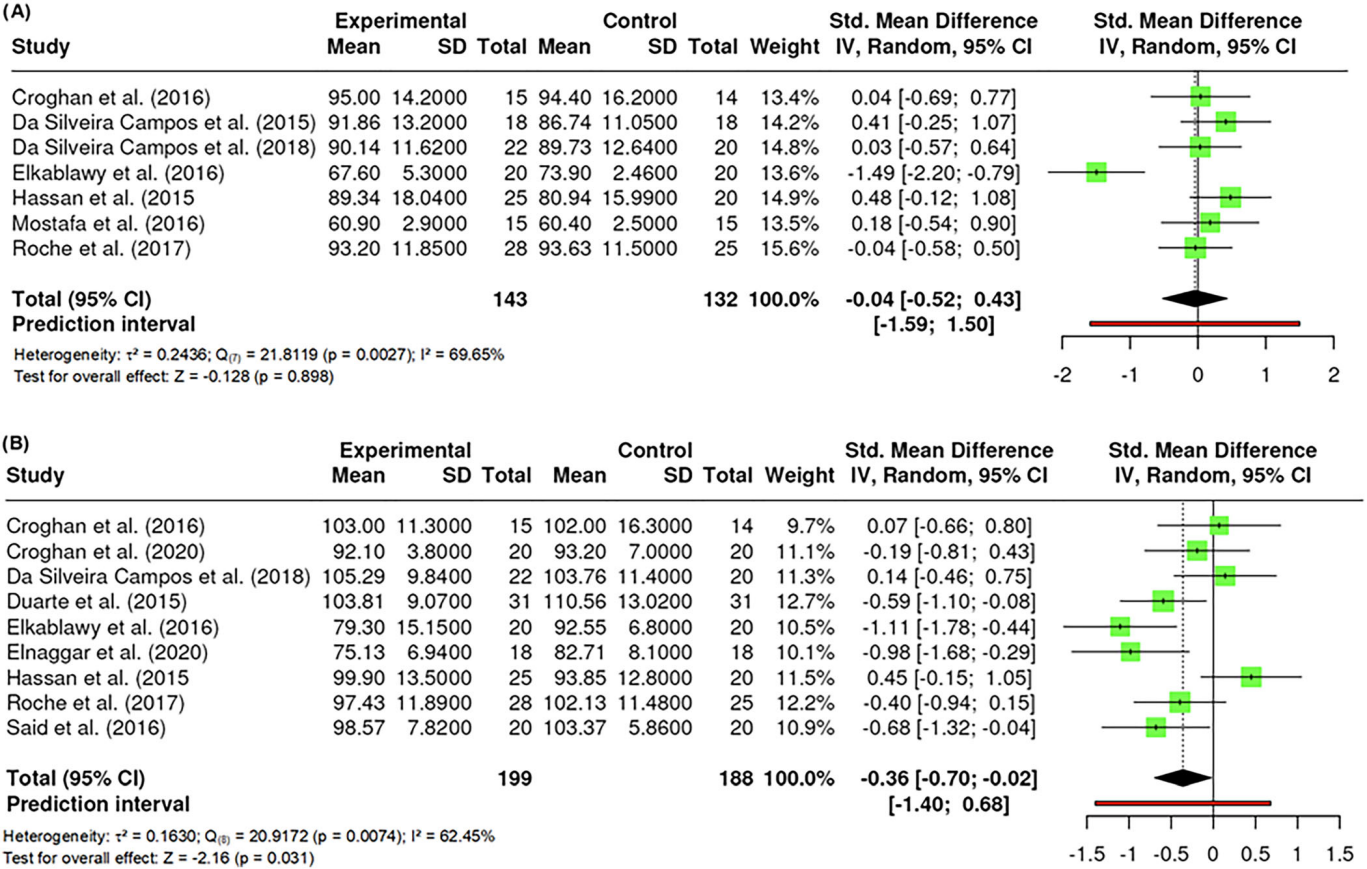
**Meta‐analysis using a random effects model of selected studies relating to weight (A) and waist circumference (B) assessed in the studies included.** The plot shows the standardized mean differences (SMD) and corresponding 95% confidence intervals (CI) for each study included in the meta‐analysis. The vertical dashed line represents the overall estimated effect size. Horizontal lines indicate the 95% CI for each study, with squares representing the individual study estimates, where the size of each square is proportional to the weight of the study in the analysis. The rhombus at the bottom represents the overall weighted effect size. The figure was generated using RevMan software.

A total of k = 8 studies performed an assessment of participants' weights measured in kilograms. The effect size varied between −1.4945 and 0.4808, with the majority of the estimates being positive (75%). The results were shown to be heterogeneous (Q _(7)_ = 21.8119, p = 0.0027, τ^2^ = 0.2436, I^2^ = 69.65%). Neither the Begg and Mazumdar Rank nor Egger's Regression indicated any funnel plot asymmetry (p = 0.9049 and p = 0.4479, respectively). However, the Fail‐Safe N (0.00; p = 0.428) suggested that there is no convincing evidence of a lack of robustness against publication bias. No difference in weight was found (Z = −0.128; p = 0.898) (Figure 2A). Given the observed variability in the wavelengths employed by the researchers, various sub‐analyses were conducted to assess potential distinctions based on wavelengths. Nevertheless, no statistically significant differences were identified in any of the examined cases.

##### Waist circumference

A total of k = 9 studies were included in the analysis of participants' waist circumferences measured in cm. The observed standardized mean differences ranged from −1.1060 to 0.4504, being negative (67%) for the majority of them. The results were shown to be heterogeneous (Q _(8)_ = 20.9172, p = 0.0074, τ^2^ = 0.1630, I^2^ = 62.4453%). Neither the Begg and Mazumdar Rank (p = 0.4767), Egger's Regression (p = 0.7584) nor Fail‐Safe N (33.00; p < 0.001) indicated any funnel plot asymmetry, suggesting robustness against publication bias. Significant differences were found in waist circumference (Z = −2.16; p = 0.031) (Figure 2B).

##### Body mass index

**FIGURE 3:**
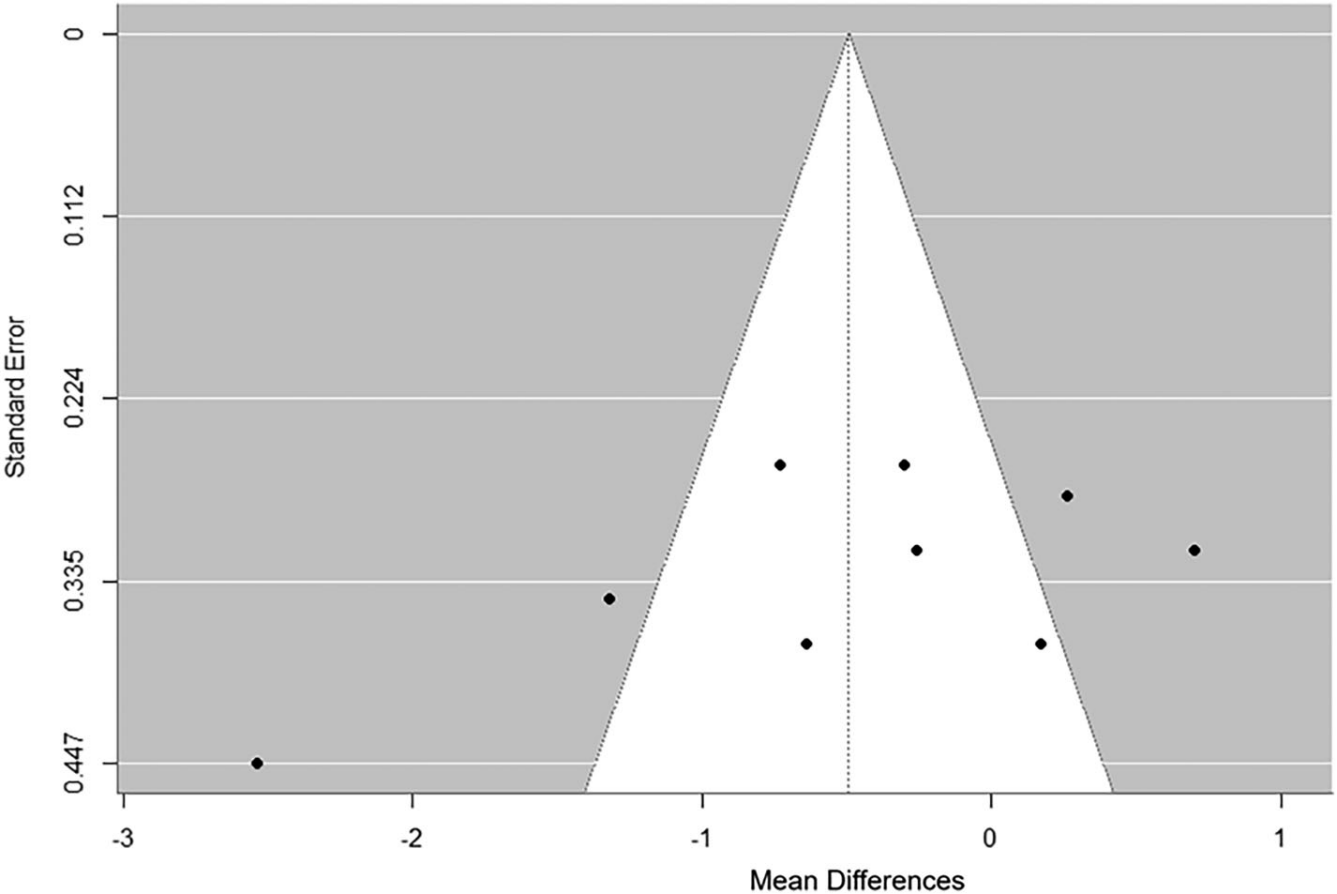
**Funnel plot of standard error (SE) by mean difference (MD) for assessment of publication bias.** Each black circle denotes a study included in the meta‐analysis (k = 11). The dashed vertical line represents the overall effect calculated with the random‐effects model. The figure was generated using R software v 3.1.

**FIGURE 4:**
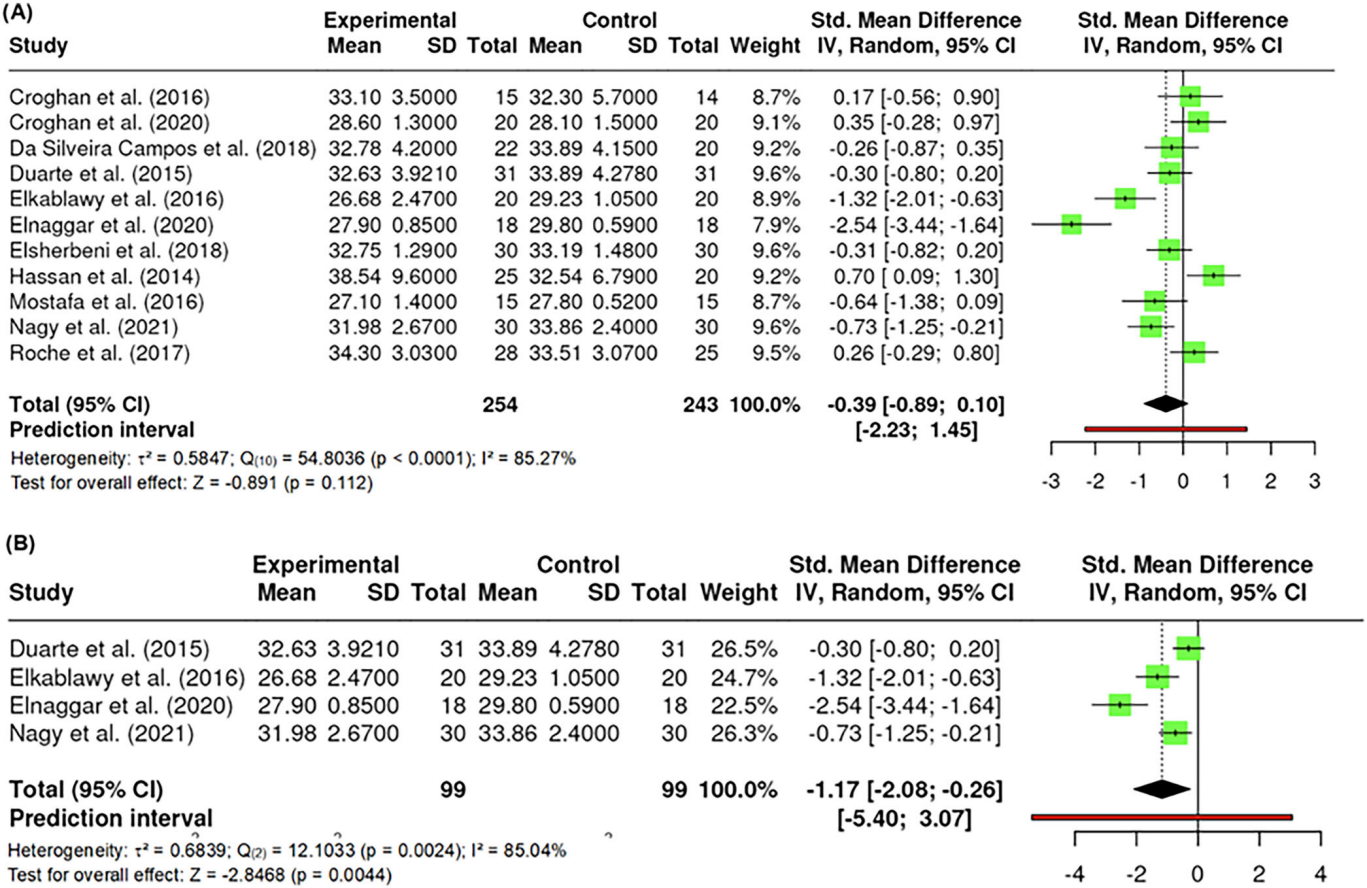
**Meta‐analysis using a random effects model of selected studies relating to BMI (A; 0.112) and BMI with the utilization of wavelengths in the 600 nm range (635 and 660 nm) (B; p = 0.0044) assessed in the studies included.** The plot shows the SMD and corresponding 95% CI for each study included in the meta‐analysis. The vertical dashed line represents the overall estimated effect size. Horizontal lines indicate the 95% CI for each study, with squares representing the individual study estimates, where the size of each square is proportional to the weight of the study in the analysis. The rhombus at the bottom represents the overall weighted effect size. The figure was generated using RevMan software.

A total of k = 11 studies were included in the analysis of participants' BMIs. The observed standardized mean differences ranged from −2.5391 to 0.6956, with the majority of estimates being negative (64%). The results were shown to be heterogeneous (Q _(10)_ = 54.8036, p < 0.0001, τ^2^ = 0.5847, I^2^ = 85.2687%). Egger's Regression test indicated funnel plot asymmetry (p = 0.0278) but not the Begg and Mazumdar Rank test (p = 0.3587) (Figure 3). However, the Fail‐Safe N (46.00; p = 0.428) suggested that there is no convincing evidence of a lack of robustness against publication bias. No difference in BMI was found (Z = −0.891; p = 0.112) (Figure 4A).

Given the observed variability in the different wavelengths utilized by the authors, various sub‐analyses were conducted, specifically focusing on wavelengths below 600 nm, at 600 nm, and at 800 nm. While the results for wavelengths below 600 nm and at 800 nm did not exhibit significance (p > 0.05), a notable effect was identified when wavelengths in the 600 nm range were employed. Three studies (k = 3) used a wavelength in the 600 nm range (635 and 660), with observed standardized mean differences ranging from −2.5391 to −0.7309, all estimates being negative. Results demonstrated heterogeneity (Q _(2)_ = 12.1033, p = 0.0024, τ^2^ = 0.6839, I^2^ = 85.0399%). While Egger's Regression test indicated funnel plot asymmetry (p = 0.0006), the Begg and Mazumdar Rank test did not (p = 0.3333). Nevertheless, the Fail‐Safe N (52.00; p = 0.001) indicated robustness against publication bias. Significant differences were observed in BMI with the utilization of wavelengths in the 600 nm range (Z = −2.8468; p = 0.0044) (Figure 4B).

##### Hip circumference

**FIGURE 5:**
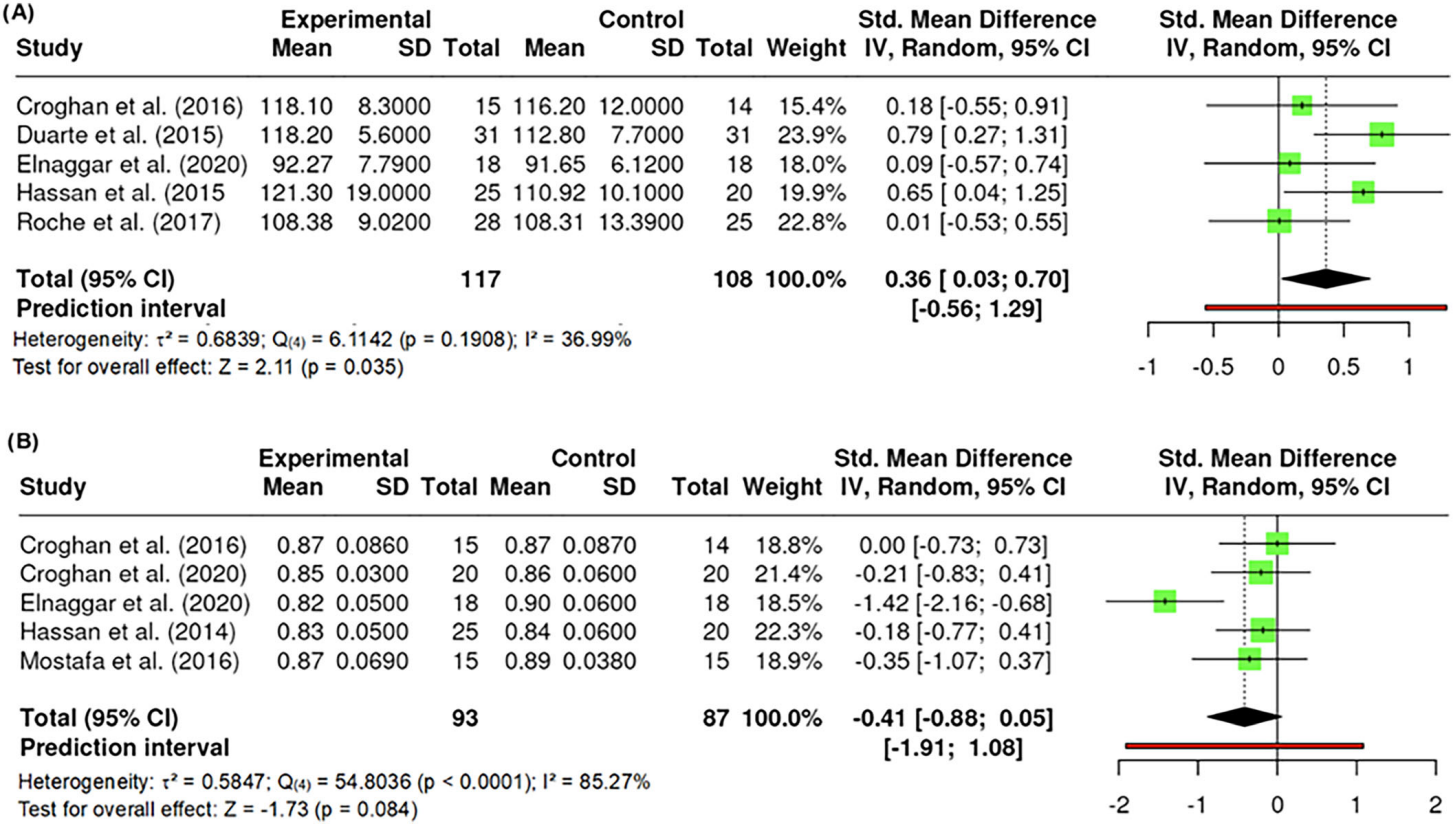
**Meta‐analysis using a random effects model of selected studies relating to hip circumference (A; p = 0.035) and waist‐to‐hip ratio (B; p = 0.084) assessed in the studies included.** The plot shows the SMD and corresponding 95% CI for each study included in the meta‐analysis. The vertical dashed line represents the overall estimated effect size. Horizontal lines indicate the 95% CI for each study, with squares representing the individual study estimates, where the size of each square is proportional to the weight of the study in the analysis. The rhombus at the bottom represents the overall weighted effect size. The figure was generated using RevMan software.

A total of k = 5 studies were included in the analysis of participants' hip circumferences, measured at the largest circumference around the buttocks. The observed standardized mean differences ranged from 0.0061 to 0.7920, with the majority of estimates being positive (100%). The results showed no significant amount of heterogeneity in the true outcomes (Q _(4)_ = 6.1142, p = 0.1908, τ^2^ = 0.0548, I^2^ = 36.9920%). Neither the Begg and Mazumdar Rank nor Egger's Regression test indicated any funnel plot asymmetry (p = 0.8167 and p = 0.4733, respectively). However, the Fail‐Safe N (8.00; p = 0.004) indicated robustness against publication bias. Significant differences were found in hip circumference (Z = 2.11; p = 0.035) (Figure 5A).

##### Waist‐to‐hip ratio

A total of k = 5 studies were included in the analysis of participants' waist‐to‐hip ratios, understood as the dimensionless ratio of the circumference of the waist to that of the hips. The observed standardized mean differences ranged from −1.4163 to 0.0000, with 80% of the estimates being negative. The results were shown to be heterogeneous (Q _(10)_ = 54.8036, p < 0.0001, τ^2^ = 0.5847, I^2^ = 85.2687%). Neither the Begg and Mazumdar Rank (p = 0.4833) nor Egger's Regression test (p = 0.4187) indicated any funnel plot asymmetry. However, the Fail‐Safe N (9.00; p = 0.004) indicated robustness against publication bias. No difference in waist‐to‐hip ratio was found (Z = −1.73; p = 0.084) (Figure 5B).

##### Neck circumference

**FIGURE 6:**
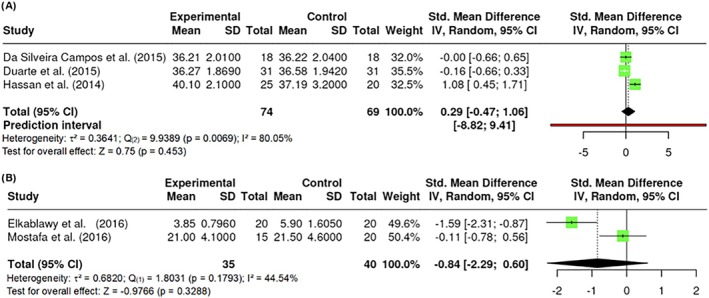
**Meta‐analysis using a random effects model of selected studies relating to neck circumference (A; p = 0.453) and skin fold (B; p = 0.3288) assessed in the studies included.** The plot shows the SMD and corresponding 95% CI for each study included in the meta‐analysis. The vertical dashed line represents the overall estimated effect size. Horizontal lines indicate the 95% CI for each study, with squares representing the individual study estimates, where the size of each square is proportional to the weight of the study in the analysis. The rhombus at the bottom represents the overall weighted effect size. The figure was generated using RevMan software.

A total of k = 3 studies performed an assessment of participants' neck circumferences. The effect size varied between −0.1642 and 1.0816, with the majority of the estimates being negative (67%). The results were shown to be heterogeneous (Q _(2)_ = 9.9389, p = 0.0069, τ^2^ = 0.3641, I^2^ = 80.0479%). Neither the Begg and Mazumdar Rank nor Egger's Regression indicated any funnel plot asymmetry (p = 1.0000 and p = 0.5764, respectively). However, the Fail‐Safe N (0.00; p = 0.059) suggested that there is no convincing evidence of a lack of robustness against publication bias. No difference in neck circumference was found (Z = 0.75; p = 0.453) (Figure 6A). It is also noteworthy to mention that all studies applied 800 nm wavelengths.

##### Skin fold

A total of k = 2 studies were included in the analysis of participants' skin folds, understood as a fold of skin formed by pinching or compressing the skin and subcutaneous layers. The observed standardized mean differences ranged from −2.2349 to 0.7484. The results showed no significant amount of heterogeneity in the true outcomes (Q _(1)_ = 1.8031, p = 0.1793, τ^2^ = 0.6820, I^2^ = 44.54%). The Fail‐Safe N (2.00; p < 0.001) indicated robustness against publication bias. No difference in skin fold was found (Z = −0.9766; p = 0.3288) (Figure 6B).

##### Lean mass

**FIGURE 7:**
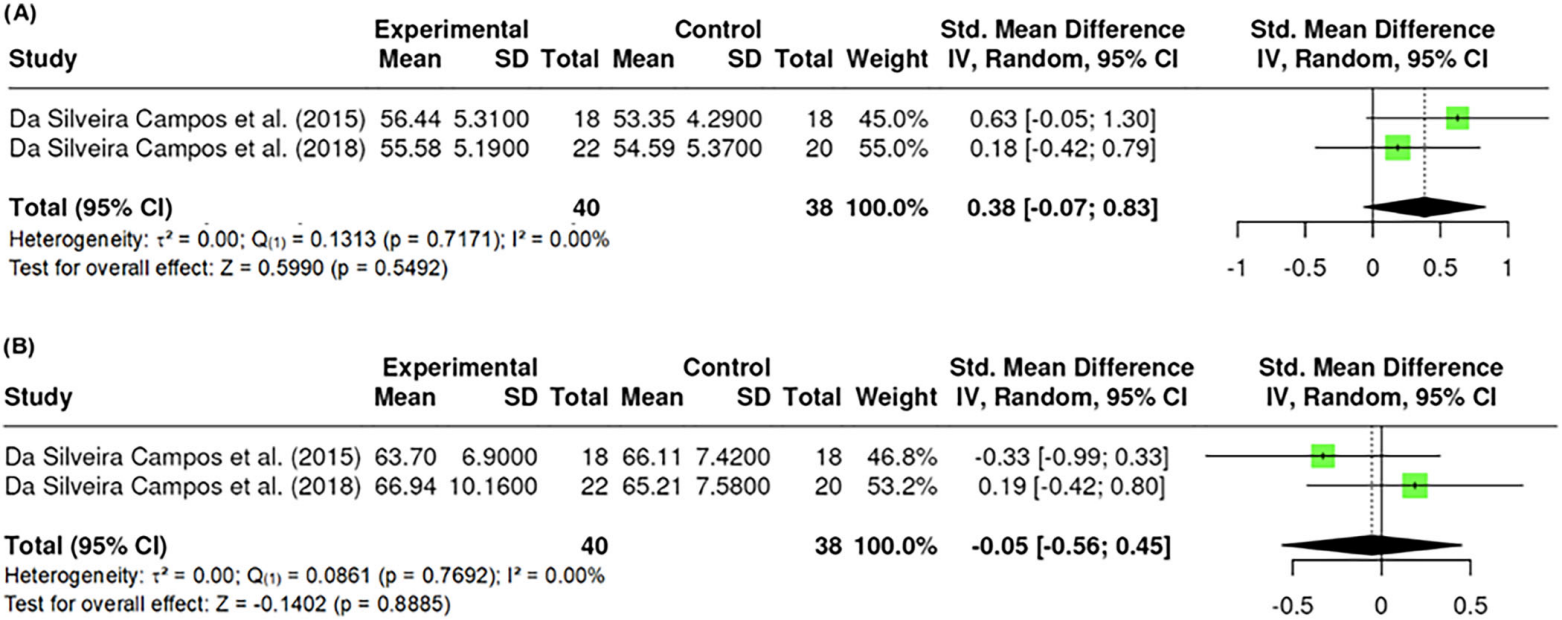
**Meta‐analysis using a random effects model of selected studies relating to lean mass in kg (A; p = 0.5492) and in % (B; p = 0.8885) assessed in the studies included.** The plot shows the SMD and corresponding 95% CI for each study included in the meta‐analysis. The vertical dashed line represents the overall estimated effect size. Horizontal lines indicate the 95% CI for each study, with squares representing the individual study estimates, where the size of each square is proportional to the weight of the study in the analysis. The rhombus at the bottom represents the overall weighted effect size. The figure was generated using RevMan software.

Lean mass was measured in the studies by the %, or kg, of lean mass in the participants' bodies. A total of k = 2 studies included a measurement of lean mass by kg. The observed standardized mean differences ranged from −11.7222 to 10.1571. The results showed no significant amount of heterogeneity in the true outcomes (Q _(1)_ = 0.1313, p = 0.7171, τ^2^ = 0, I^2^ = 0.00%). Similarly, the Fail‐Safe N (0.00; p = 0.0945) suggested that there is no convincing evidence of a lack of robustness against publication bias. On the other hand, a total of k = 2 studies included a measurement of lean mass by % in the body. The observed standardized mean differences ranged from −4.8507 to 9.1206. The results showed no significant amount of heterogeneity in the true outcomes (Q _(1)_ = 0.0861, p = 0.7692, τ^2^ = 0, I^2^ = 0.00%). However, the Fail‐Safe N (0.00; p = 0.8335) suggested that there is no convincing evidence of a lack of robustness against publication bias. No difference in lean mass was found in kg (Z = 0.5990; p = 0.5492) (Figure 7A) or % (Z = −0.1402; p = 0.8885) (Figure 7B).

##### Fat mass

**FIGURE 8:**
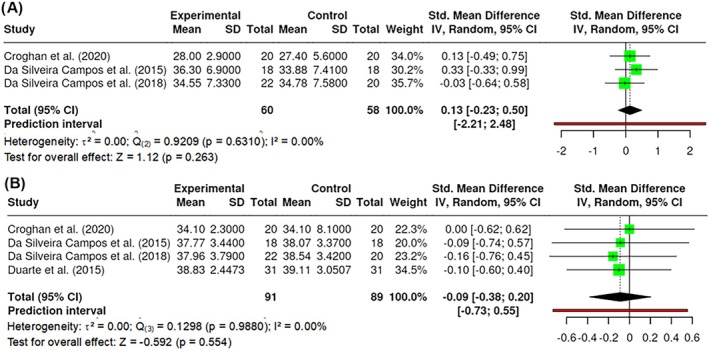
**Meta‐analysis using a random effects model of selected studies relating to fat mass in kg (A; p = 0.263) and in % (B; p = 0.554) assessed in the studies included.** The plot shows the SMD and corresponding 95% CI for each study included in the meta‐analysis. The vertical dashed line represents the overall estimated effect size. Horizontal lines indicate the 95% CI for each study, with squares representing the individual study estimates, where the size of each square is proportional to the weight of the study in the analysis. The rhombus at the bottom represents the overall weighted effect size. The figure was generated using RevMan software.

Fat mass was measured in the studies by the %, or kg, of fat mass in the participants' bodies. A total of k = 3 studies included a measurement of fat mass by kg. The observed standardized mean differences ranged from −0.1572 to 0.0000, with the majority of estimates being negative (75%). According to heterogeneity statistics, there was no significant amount of heterogeneity in the true outcomes (Q _(2)_ = 0.9209, p = 0.6310, τ^2^ = 0.0000, I^2^ = 0.00%). Neither the Begg and Mazumdar Rank nor Egger's Regression indicated any funnel plot asymmetry (p = 1.0000 and p = 0.777, respectively). However, the Fail‐Safe N (0.00; p = 0.127) suggested that there is no convincing evidence of a lack of robustness against publication bias. A total of k = 4 studies included a measurement of fat mass by % in the body. The observed standardized mean differences ranged from −4.8507 to 9.1206. The results showed no significant amount of heterogeneity in the true outcomes (Q _(3)_ = 0.1298, p = 0.9880, τ^2^ = 0.0000, I^2^ = 0.00%). However, the Fail‐Safe N (0.00; p = 0.281) suggested that there is no convincing evidence of a lack of robustness against publication bias. No difference in fat mass was found in kg (Z = 1.12; p = 0.263) (Figure 8A) nor % (Z = −0.592; p = 0.554) (Figure 8B). All reported studies applied 800 nm wavelength.

##### Visceral fat

**FIGURE 9:**
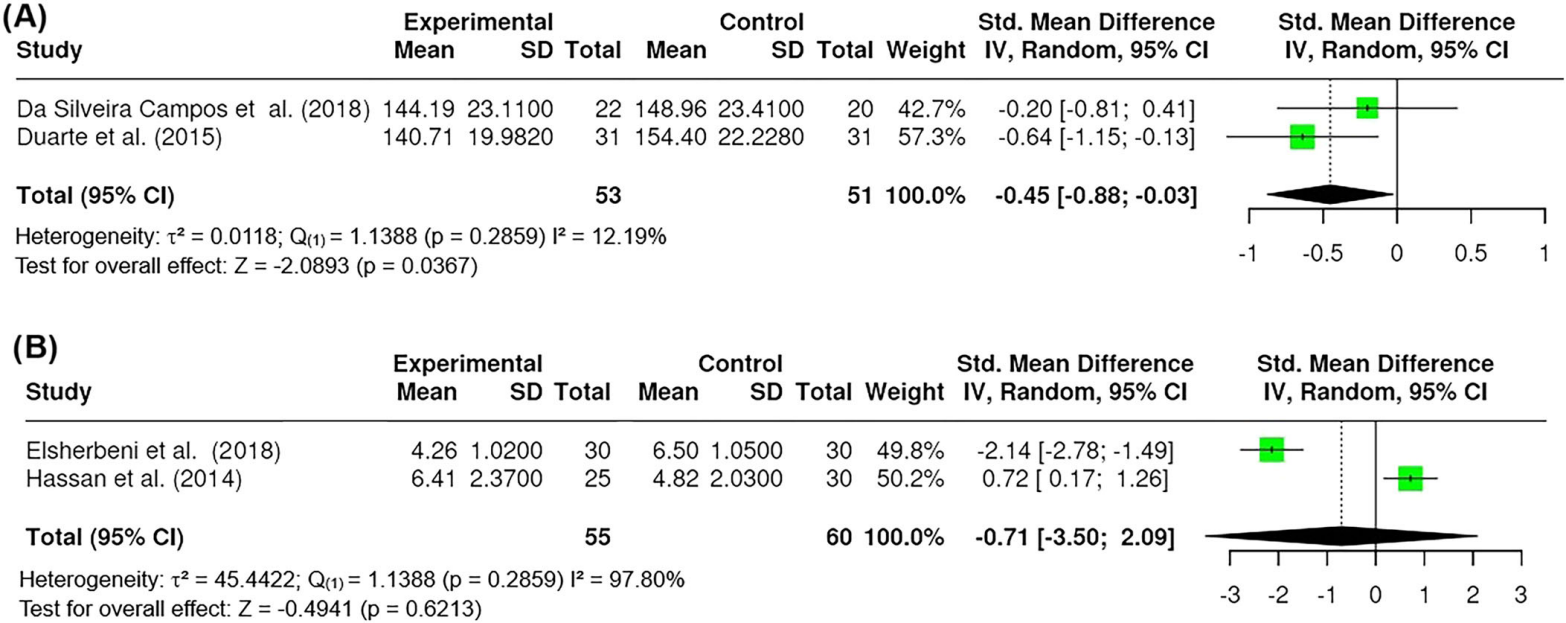
**Meta‐analysis using a random effects model of selected studies relating to visceral fat in cm** ^**2**^ **(A; p = 0.5370) or USVF (B; p = 0.6213) assessed in the studies included.** The plot shows the SMD and corresponding 95% CI for each study included in the meta‐analysis. The vertical dashed line represents the overall estimated effect size. Horizontal lines indicate the 95% CI for each study, with squares representing the individual study estimates, where the size of each square is proportional to the weight of the study in the analysis. The rhombus at the bottom represents the overall weighted effect size. The figure was generated using RevMan software.

Visceral fat was measured in the studies by cm^2^, or the visceral fat at the umbilicus (USVF) in cm. The USVF corresponded to the measurement from the internal surface of the abdominal rectus muscle and the posterior aortic wall in the abdominal midline during expiration. A total of k = 2 studies included a measurement of visceral fat by cm^2^. The observed standardized mean differences ranged from 0.8801 to −0.0281. The results showed no significant amount of heterogeneity in the true outcomes (Q _(1)_ = 1.1388, p = 0.2859, τ^2^ = 0.0118, I^2^ = 12.19%). A total of k = 2 studies included a measurement of USVF. The observed standardized mean differences ranged from −3.5092 to 2.0962. The results showed a significant amount of heterogeneity in the true outcomes (Q _(1)_ = 45.4422, p = 0.0001, τ^2^ = 3.9998, I^2^ = 97.80%). A significant difference in visceral fat was found in cm^2^ (Z = −2.0893; p = 0.0367) (Figure 9A) but no significant difference was observed in USVF (Z = −0.4941; p = 0.6213) (Figure 9B). All reported studies applied 800 nm wavelengths.

#### 3.1.2 Physiological variables

##### Fasting glucose

**FIGURE 10:**
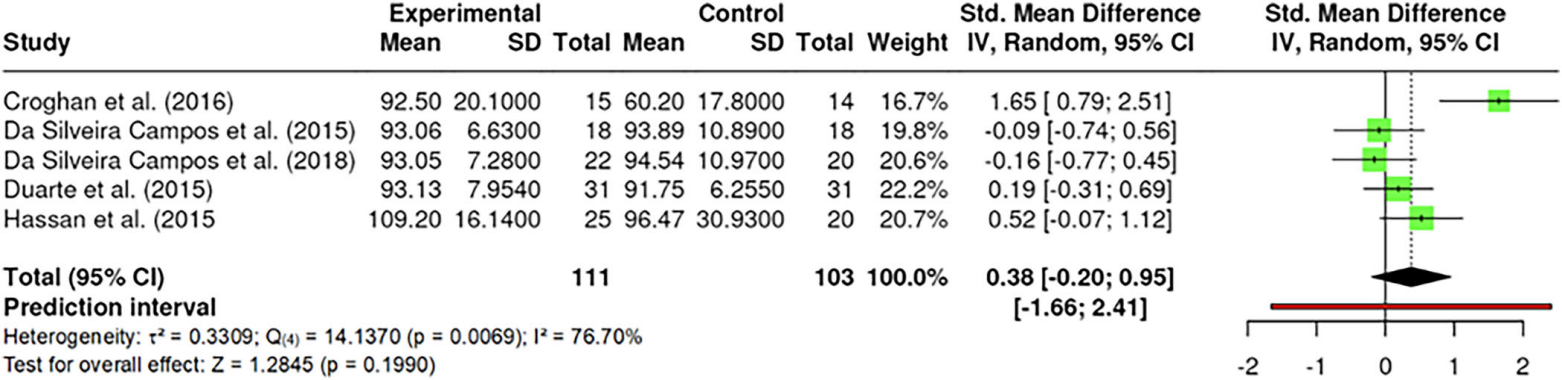
**Meta‐analysis using a random effects model of selected studies relating to fasting glucose (p = 0.1990) assessed in the studies included.** The plot shows the SMD and corresponding 95% CI for each study included in the meta‐analysis. The vertical dashed line represents the overall estimated effect size. Horizontal lines indicate the 95% CI for each study, with squares representing the individual study estimates, where the size of each square is proportional to the weight of the study in the analysis. The rhombus at the bottom represents the overall weighted effect size. The figure was generated using RevMan software.

Fasting glucose was evaluated by the levels of glucose in the blood of participants who had fasted for at least 8 hours. A total of k = 5 studies were included. The observed standardized mean differences ranged from −0.1586 to 1.6499, with the majority of estimates being positive (60%). The results were shown to be heterogeneous (Q _(4)_ = 14.1370, p = 0.0069, τ^2^ = 0.3309, I^2^ = 76.6968%). Neither the Begg and Mazumdar Rank (p = 0.8167) nor Egger's Regression test (p = 0.0625) indicated any funnel plot asymmetry. Nevertheless, the Fail‐Safe N (7.00; p = 0.007) suggested robustness against publication bias. No significant difference in fasting glucose was detected (Z = 1.2845, p = 0.1990) (Figure 10). A sub‐analysis excluding the study from Croghan et al^7^ which employed a different wavelength, was conducted. However, no significant differences were observed at a range from 808 to 843 nm (p > 0.05).

##### Lipids

**FIGURE 11:**
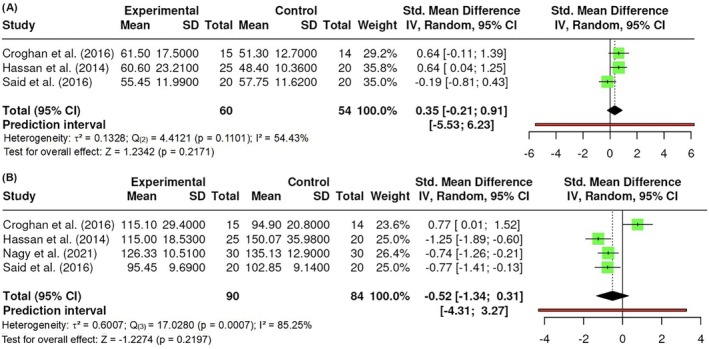
**Meta‐analysis using a random effects model of selected studies relating to lipids, specifically to HDL (A; p = 0.2171) and LDL (B; p = 0.2171) assessed in the studies included.** The plot shows the SMD and corresponding 95% CI for each study included in the meta‐analysis. The vertical dashed line represents the overall estimated effect size. Horizontal lines indicate the 95% CI for each study, with squares representing the individual study estimates, where the size of each square is proportional to the weight of the study in the analysis. The rhombus at the bottom represents the overall weighted effect size. The figure was generated using RevMan software.

The lipid variables were LDL, high‐density lipoprotein (HDL), and triglycerides. A total of k = 3 studies were included in the analysis of HDL, and the observed standardized mean differences ranged from −0.1909 to 0.6447, with the majority of estimates being positive (67%). The results were shown to be heterogeneous (Q _(2)_ = 4.4121, p = 0.1101, τ^2^ = 0.1328, I^2^ = 54.4344%). Neither the Begg and Mazumdar Rank (p = 1.000) nor Egger's Regression test (p = 0.6864) indicated any funnel plot asymmetry. However, the Fail‐Safe N (1.00; p = 0.033) indicated robustness against publication bias. A total of k = 4 studies were included in the analysis of LDL, and the observed standardized mean differences ranged from −1.2468 to 0.7662, with the majority of estimates being negative (75%). The results were shown to be heterogeneous (Q _(3)_ = 17.0280, p = 0.0007, τ^2^ = 0.6007, I^2^ = 85.2497%). Neither the Begg and Mazumdar Rank (p = 0.3333) nor Egger's Regression test (p = 0.1979) indicated any funnel plot asymmetry. However, the Fail‐Safe N (14.00; p < 0.001) indicated robustness against publication bias. No difference in HDL (Z = 1.2342, p = 0.2171) (Figure 11A) or LDL was found (Z = −1.2274, p = 0.2197) (Figure 11B).

**FIGURE 12:**
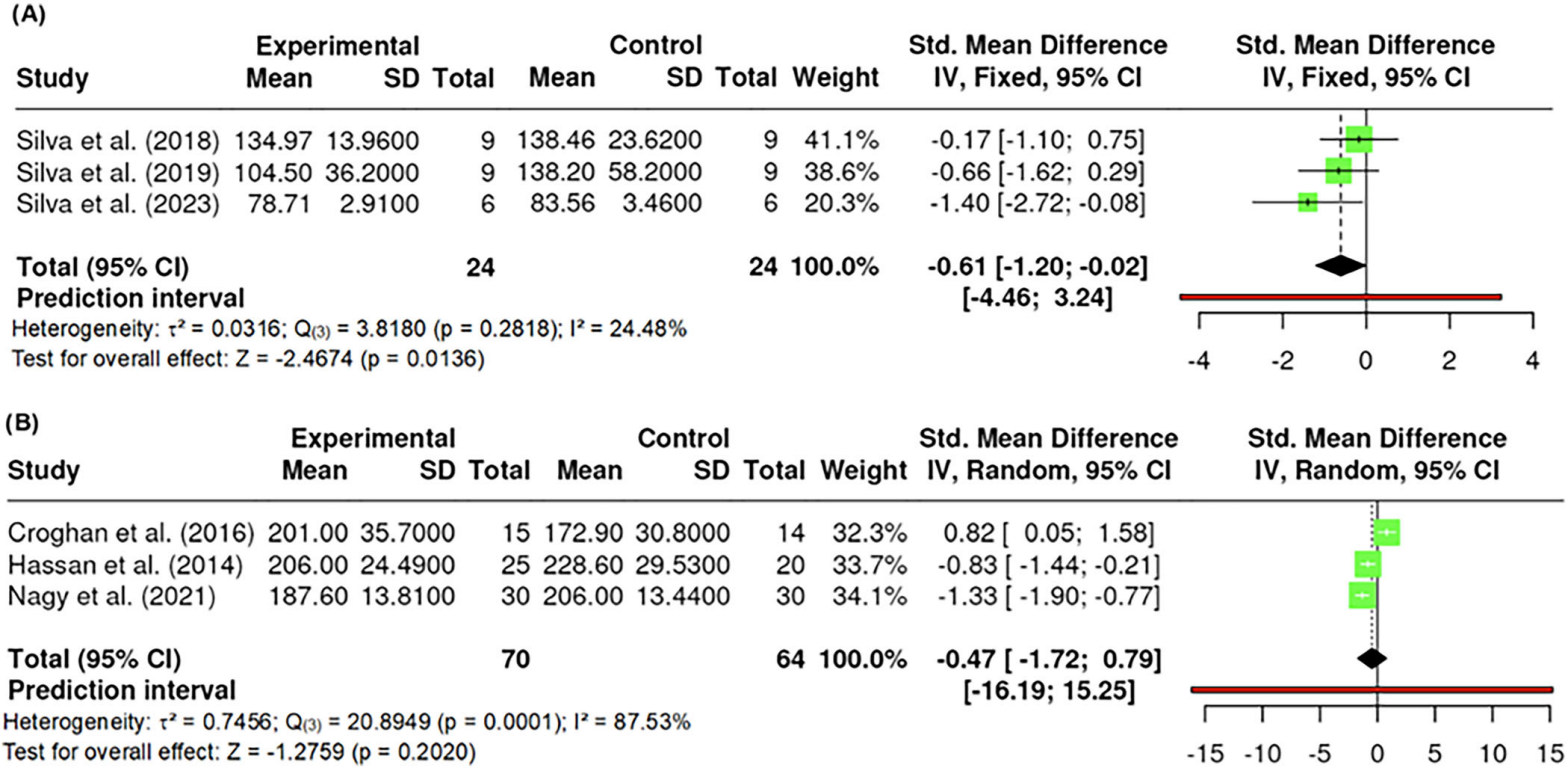
**Meta‐analysis using a random effects model of selected studies relating to triglycerides (A; p = 0.0136) and total cholesterol (B; p = 0.2020) assessed in the studies included.** The plot shows the SMD and corresponding 95% CI for each study included in the meta‐analysis. The vertical dashed line represents the overall estimated effect size. Horizontal lines indicate the 95% CI for each study, with squares representing the individual study estimates, where the size of each square is proportional to the weight of the study in the analysis. The rhombus at the bottom represents the overall weighted effect size. The figure was generated using RevMan software.

A total of k = 4 studies were included in the analysis of triglycerides, with an observed standardized mean difference that ranged from 0.0061 to 0.7920, with all of the estimates being positive (100%). The results showed no significant amount of heterogeneity in the true outcomes (Q _(3)_ = 3.8180, p = 0.2818, τ^2^ = 0.0316, I^2^ = 24.4795%). Neither the Begg and Mazumdar Rank nor Egger's Regression test indicated any funnel plot asymmetry (p = 0.7500 and p = 0.4266, respectively). However, the Fail‐Safe N (8.00; p = 0.003) indicated robustness against publication bias. Significant differences were found in triglycerides (Z = −2.4674, p = 0.0136) (Figure 12A).

##### Total cholesterol

A total of k = 4 studies were included, and the observed standardized mean differences ranged from −1.3328 to 0.8169, with the majority of estimates being negative (75%). The results were shown to be heterogeneous (Q _(3)_ = 20.8949, p = 0.0001, τ^2^ = 0.7456, I^2^ = 87.5323%). Egger's Regression test indicated funnel plot asymmetry (p < 0.0001) but not the Begg and Mazumdar Rank test (p = 0.3333). However, the Fail‐Safe N (20.00; p < 0.001) indicated robustness against publication bias. No difference in total cholesterol was found (Z = −1.2759, p = 0.2020) (Figure 12B).

##### Insulin

**FIGURE 13:**
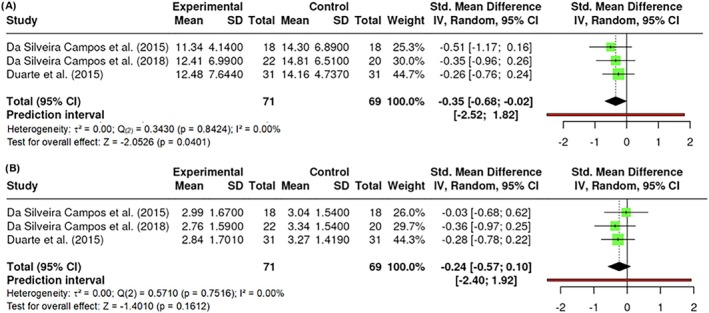
**Meta‐analysis using a random effects model of selected studies relating to insulin (A; p = 0.0401) and HOMA‐IR (B; p = 0.1612) assessed in the studies included.** The plot shows the SMD and corresponding 95% CI for each study included in the meta‐analysis. The vertical dashed line represents the overall estimated effect size. Horizontal lines indicate the 95% CI for each study, with squares representing the individual study estimates, where the size of each square is proportional to the weight of the study in the analysis. The rhombus at the bottom represents the overall weighted effect size. The figure was generated using RevMan software.

A total of k = 3 studies were included in the analysis with an observed standardized mean difference that ranged from −0.5092 to −0.2609, with all of the estimates being negatives (100%). The results showed no significant amount of heterogeneity in the true outcomes (Q _(2)_ = 0.3430, p = 0.8424, τ^2^ = 0.0000, I^2^ = 0.0000%). Neither the Begg and Mazumdar Rank nor Egger's Regression test indicated any funnel plot asymmetry (p = 0.3333 and p = 0.5825, respectively). However, the Fail‐Safe N (2.00; p = 0.018) indicated robustness against publication bias. Significant differences were found in insulin (Z = −2.0526, p = 0.0401). All reported studies applied 800 nm wavelengths (Figure 13A).

HOMA‐IR (homeostasis model assessment‐insulin resistant).

The HOMA‐IR (homeostasis model assessment‐insulin resistant) is widely used as an indirect method for quantifying insulin resistance and pancreatic β‐cell function. A total of k = 3 studies performed an assessment of participants' HOMA‐IR levels. The effect size varied from −0.3633 to −0.0304, with all of the estimates being negative (100%). The results showed no significant amount of heterogeneity in the true outcomes (Q _(2)_ = 0.5710, p = 0.7516, τ^2^ = 0.0000, I^2^ = 0.0000%). Neither the Begg and Mazumdar Rank nor Egger's Regression indicated any funnel plot asymmetry (p = 1.0000 and p = 0.7145, respectively). However, the Fail‐Safe N (0.00; p = 0.089) suggested that there is no convincing evidence of a lack of robustness against publication bias. No difference in HOMA‐IR was found (Z = −1.4010, p = 0.1612) (Figure 13B). All reported studies applied 800 nm wavelengths.

##### Leptin

**FIGURE 14:**
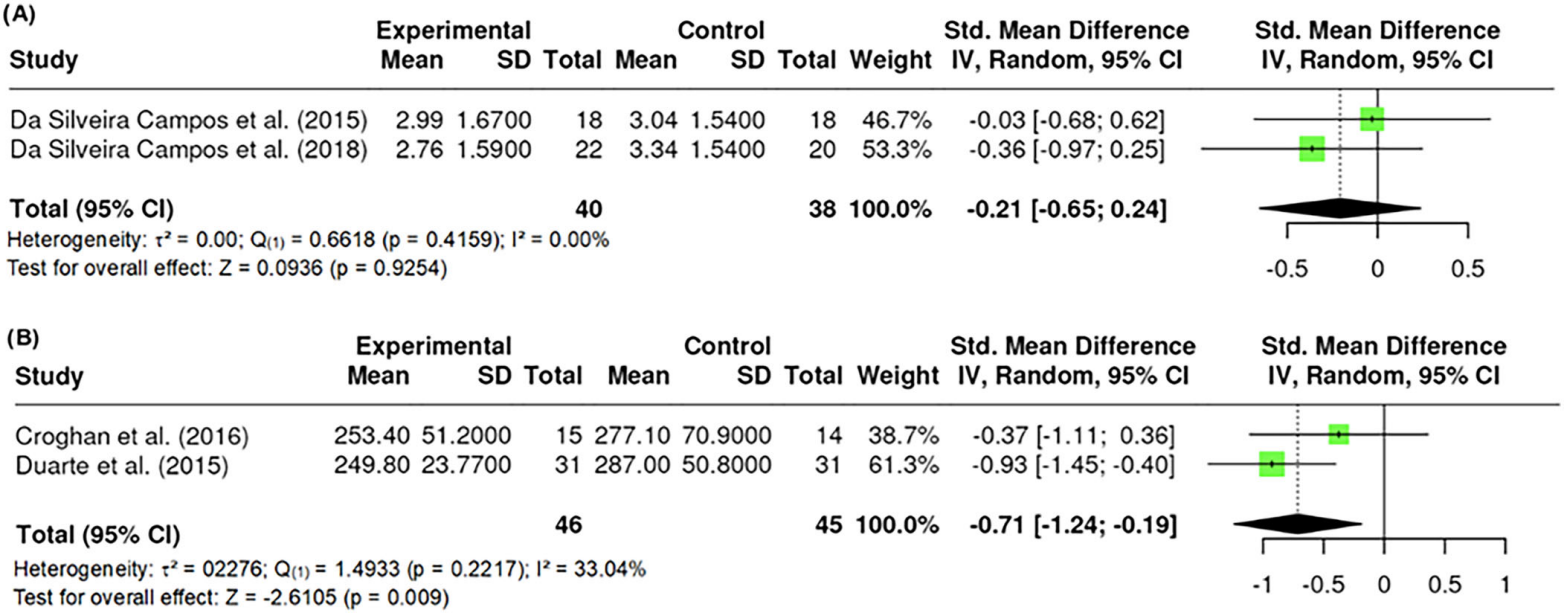
**Meta‐analysis using a random effects model of selected studies relating to leptin (A; p = 0.9254) and platelets (B; p = 0.009) assessed in the studies included.** The plot shows the SMD and corresponding 95% CI for each study included in the meta‐analysis. The vertical dashed line represents the overall estimated effect size. Horizontal lines indicate the 95% CI for each study, with squares representing the individual study estimates, where the size of each square is proportional to the weight of the study in the analysis. The rhombus at the bottom represents the overall weighted effect size. The figure was generated using RevMan software.

Leptin was measured by a total of k = 2 studies that observed standardized mean differences ranging between −0.5715 and 0.6288. The results showed no significant amount of heterogeneity in the true outcomes (Q _(1)_ = 0.6618, p = 0.4159, τ^2^ = 0, I^2^ = 0.00%). Similarly, the Fail‐Safe N (0.00; p = 0.2951) suggested that there is no convincing evidence of a lack of robustness against publication bias. No difference in leptin was found (Z = 0.0936; p = 0.9254) (Figure 14A).

##### Platelets

Platelets were measured by a total of k = 2 studies that observed standardized mean differences ranging between −1.2474 and −0.1776. The results showed no significant amount of heterogeneity in the true outcomes (Q _(1)_ = 1.4933, p = 0.2217, τ^2^ = 0.2276, I^2^ = 33.04%). However, the Fail‐Safe N (1.00; p = 0.0079) indicated robustness against publication bias. A significant difference in platelets was found (Z = −2.6105; p = 0.009) (Figure 14B).

#### 3.1.3 Psychological variables

##### Linear analogue self‐assessment

**FIGURE 15:**
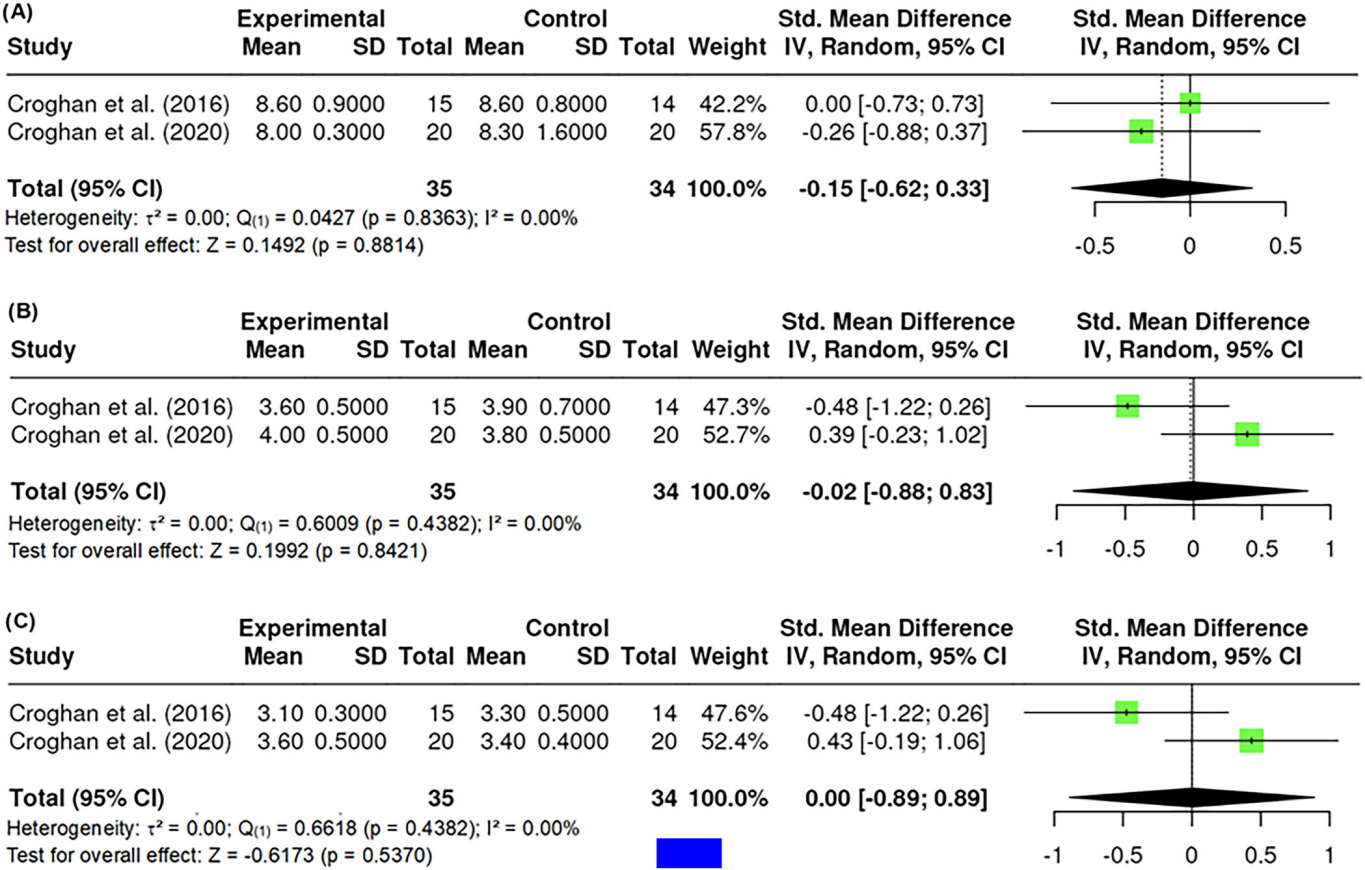
**Meta‐analysis using a random effects model of selected studies relating to quality of life assessed with the LASS (A; p = 0.8814) and body image assessed with the BASS (B; p = 0.4382) and the BASS (C; p = 0.5370) assessed in the studies included.** The plot shows the SMD and corresponding 95% CI for each study included in the meta‐analysis. The vertical dashed line represents the overall estimated effect size. Horizontal lines indicate the 95% CI for each study, with squares representing the individual study estimates, where the size of each square is proportional to the weight of the study in the analysis. The rhombus at the bottom represents the overall weighted effect size. The figure was generated using RevMan software.

The Linear Analogue Self‐Assessment (LASA) is a common instrument to measure the quality of life, considering the perceived level of functioning. LASA was measured by a total of k = 2 studies that observed standardized mean differences ranging between −1.2475 and 1.4531. The results showed no significant amount of heterogeneity in the true outcomes (Q _(1)_ = 0.0427, p = 0.8363, τ^2^ = 0, I^2^ = 0.00%). Similarly, the Fail‐Safe N (0.00; p = 0.5496) suggested that there is no convincing evidence of a lack of robustness against publication bias. No difference in LASA was found (Z = 0.1492; p = 0.8814) (Figure 15A).

##### Body image

The body image was assessed with the Body Appreciation Scale (BAS) and the Body Area Satisfaction Scale (BASS). The BAS is a test that measures participants' attitudes toward their bodies. BAS was measured by a total of k = 2 studies, in which the observed standardized mean differences ranged between −0.6600 and 0.8093. The results showed no significant amount of heterogeneity in the true outcomes (Q _(1)_ = 0.6009, p = 0.4382, τ^2^ = 0, I^2^ = 0.00%). Similarly, the Fail‐Safe N (0.00; p = 0.8763) suggested that there is no convincing evidence of a lack of robustness against publication bias. On the other hand, the Body Area Satisfaction Scale (BASS) assessed the degree of satisfaction with specific body areas and attributes. BASS was measured by a total of k = 2 studies, in which the observed standardized mean differences ranged between −0.5715 and 0.6288. The results showed no significant amount of heterogeneity in the true outcomes (Q _(1)_ = 0.6618, p = 0.4159, τ^2^ = 0, I^2^ = 0.00%). Similarly, the Fail‐Safe N (0.00; p = 0.8543) suggested that there is no convincing evidence of a lack of robustness against publication bias. No difference in BAS (Z = 0.1992; p = 0.8421) or BASS (Z = −0.6173; p = 0.5370) was found (Figure 15B and C).

Animal models.

#### 3.1.4 Body mass

**FIGURE 16:**
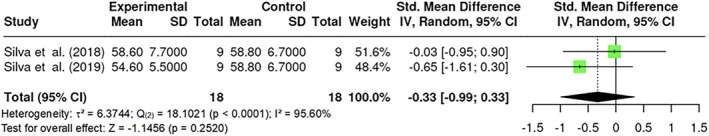
**Meta‐analysis using a random effects model of selected studies relating to body mass (p = 0.2520) in mice models assessed in the studies included.** The plot shows the SMD and corresponding 95% CI for each study included in the meta‐analysis. The vertical dashed line represents the overall estimated effect size. Horizontal lines indicate the 95% CI for each study, with squares representing the individual study estimates, where the size of each square is proportional to the weight of the study in the analysis. The rhombus at the bottom represents the overall weighted effect size. The figure was generated using RevMan software.

The assessment of body mass, measured in grams, encompassed a total of k = 3 studies conducted in mouse models. Estimated effect sizes varied from −5.3126 to 1.3931, with significant heterogeneity observed (Q _(2)_ = 18.1021, p < 0.0001, τ^2^ = 8.1493, I^2^ = 95.60%). Neither Egger's test (p = 0.1227) nor Begg and Mazumdar's rank correlation test (p = 0.3333) showed evidence of publication bias or significant asymmetry. However, the overall estimate was not statistically significant (Z = −1.1456, p = 0.2520), indicating that PBM did not significantly affect body mass in these models (Figure 16).

#### 3.1.5 Adipocyte area analysis

**FIGURE 17:**
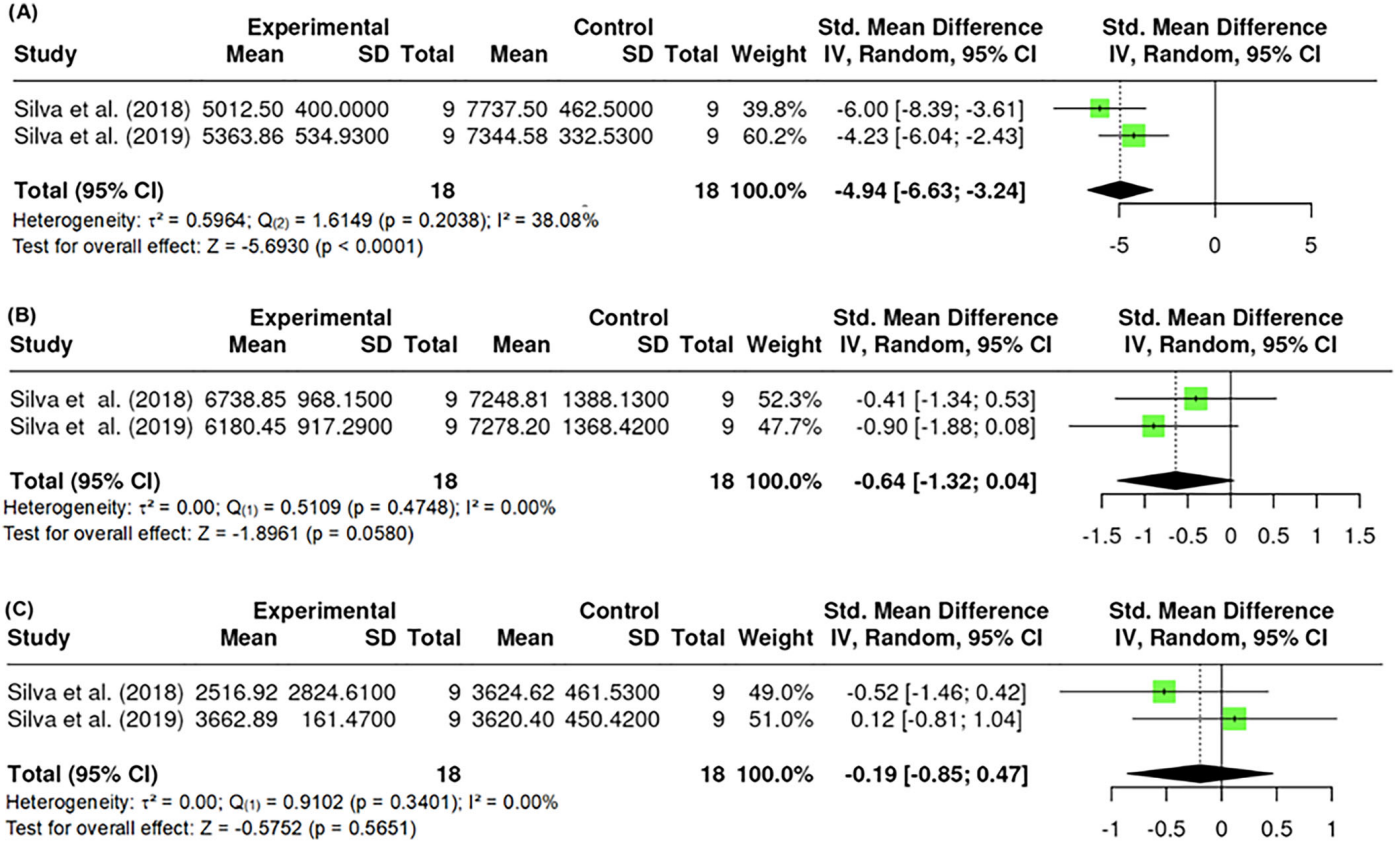
**Meta‐analysis using a random effects model of selected studies relating to epididymal adipocyte area (A; p < 0.0001), retroperitoneal adipocyte area (B; p = 0.0580), and mesenteric adipocyte area (C; p = 0.5651) in mice models assessed in the studies included.** The plot shows the SMD and corresponding 95% CI for each study included in the meta‐analysis. The vertical dashed line represents the overall estimated effect size. Horizontal lines indicate the 95% CI for each study, with squares representing the individual study estimates, where the size of each square is proportional to the weight of the study in the analysis. The rhombus at the bottom represents the overall weighted effect size. The figure was generated using RevMan software.

The analysis of the epididymal adipocyte area, expressed in μm^2^, included k = 2 studies. The estimated effect sizes ranged from −6.6861 to −3.2614, with moderate heterogeneity (Q _(1)_ = 1.6149, p = 0.2038, τ^2^ = 0.5964, I^2^ = 38.08%). The Begg and Mazumdar rank correlation test (p = 1.0000) indicated no evidence of publication bias or significant asymmetry. This analysis revealed a statistically significant impact of PBM (Z = −5.6930, p < 0.0001) on the epididymal adipocyte area (Figure 17A).

For the retroperitoneal adipocyte area, also measured in μm^2^, k = 2 studies were analyzed, yielding estimated effect sizes from −1.3215 to 0.0219 and no heterogeneity (Q _(1)_ = 0.5109, p = 0.4748, τ^2^ = 0, I^2^ = 0.00%). The Begg and Mazumdar rank correlation test (p = 1.0000) indicated no evidence of publication bias or significant asymmetry. The overall estimate was not statistically significant (Z = −1.8961, p = 0.0580), suggesting no meaningful effect of PBM on this parameter (Figure 17B).

Similarly, the mesenteric adipocyte area, also expressed in μm^2^, included k = 2 studies. Effect sizes ranged from −0.8501 to 0.4643, with no heterogeneity (Q _(1)_ = 0.9102, p = 0.3401, τ^2^ = 0, I^2^ = 0.00%). The Begg and Mazumdar rank correlation test (p = 1.0000) indicated no evidence of publication bias or significant asymmetry. The overall estimate was not statistically significant (Z = −0.5752, p = 0.5651), indicating no significant effect of PBM on the mesenteric adipocyte area (Figure 17C).

#### 3.1.6 Lipid profile analysis

**FIGURE 18:**
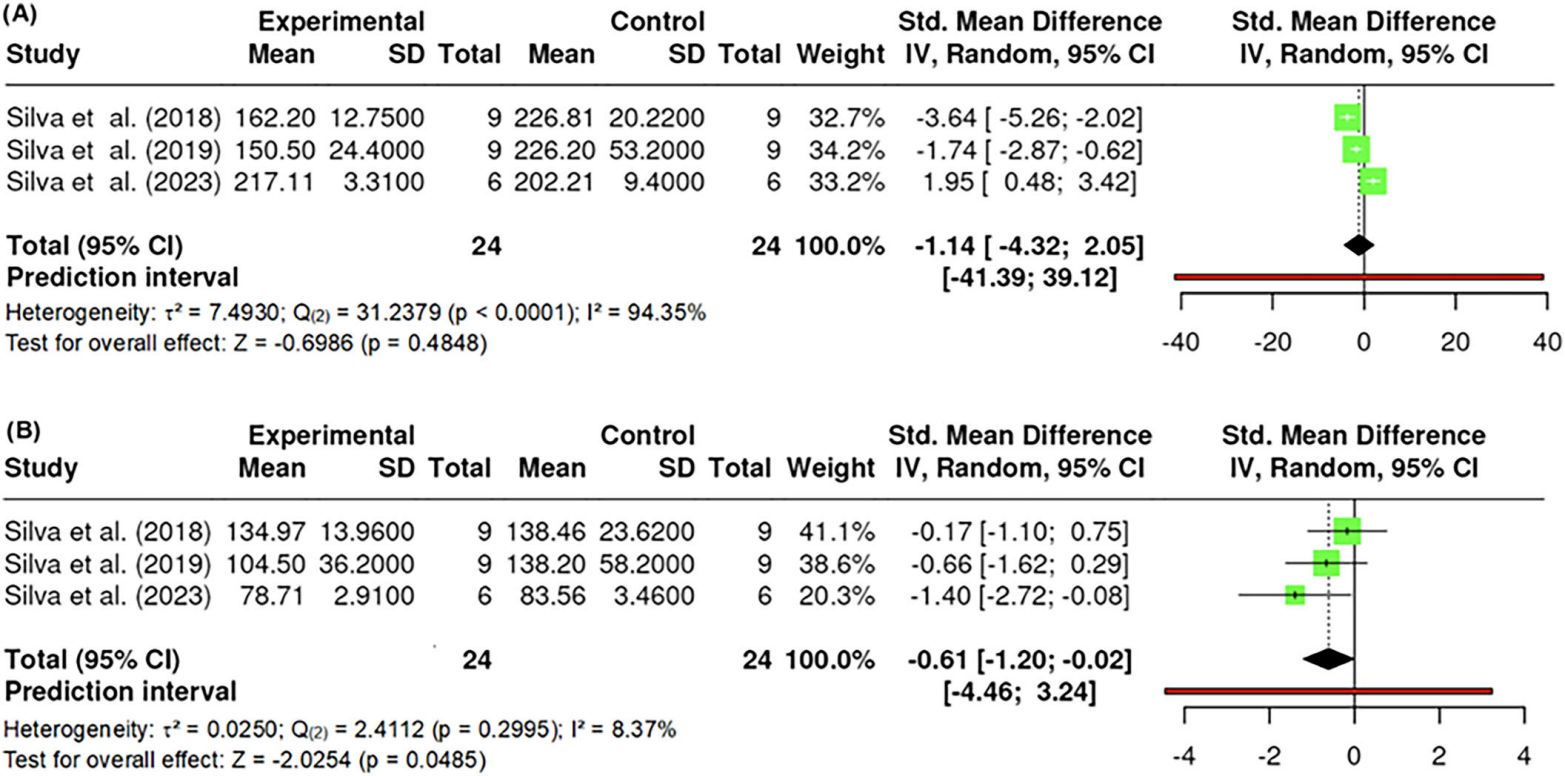
**Meta‐analysis using a random effects model of selected studies relating to cholesterol (A; p = 0.4848) and triglyceride levels (B; p = 0.0485) in mice models assessed in the studies included.** The plot shows the SMD and corresponding 95% CI for each study included in the meta‐analysis. The vertical dashed line represents the overall estimated effect size. Horizontal lines indicate the 95% CI for each study, with squares representing the individual study estimates, where the size of each square is proportional to the study's weight in the analysis. The rhombus at the bottom represents the overall weighted effect size. The figure was generated using RevMan software.

In the cholesterol assessment, k = 3 studies were included. Estimated effect sizes ranged from −4.3292 to 2.0539, exhibiting high heterogeneity (Q _(2)_ = 31.2379, p < 0.0001, τ^2^ = 7.4930, I^2^ = 94.35%). Neither Egger's test (p = 0.9977) nor Begg and Mazumdar's rank correlation test (p = 1.0000) showed evidence of publication bias or significant asymmetry. The overall estimate was not statistically significant (Z = −0.6986, p = 0.4848), suggesting that PBM had no significant effect on cholesterol levels (Figure 18A).

Conversely, the analysis of triglycerides, also involving k = 3 studies, showed estimated effect sizes from −4.3292 to 2.0539, with low heterogeneity (Q _(2)_ = 2.4112, p = 0.2995, τ^2^ = 0.0250, I^2^ = 8.37%). Neither Egger's test (p = 0.2683) nor Begg and Mazumdar's rank correlation test (p = 0.3333) showed evidence of publication bias or significant asymmetry. Despite this, the overall estimate was statistically significant (Z = −2.0254, p = 0.0485), indicating a notable impact of PBM on triglyceride levels (Figure 18B).

#### 3.1.7 Glucose and insulin analysis

**FIGURE 19:**
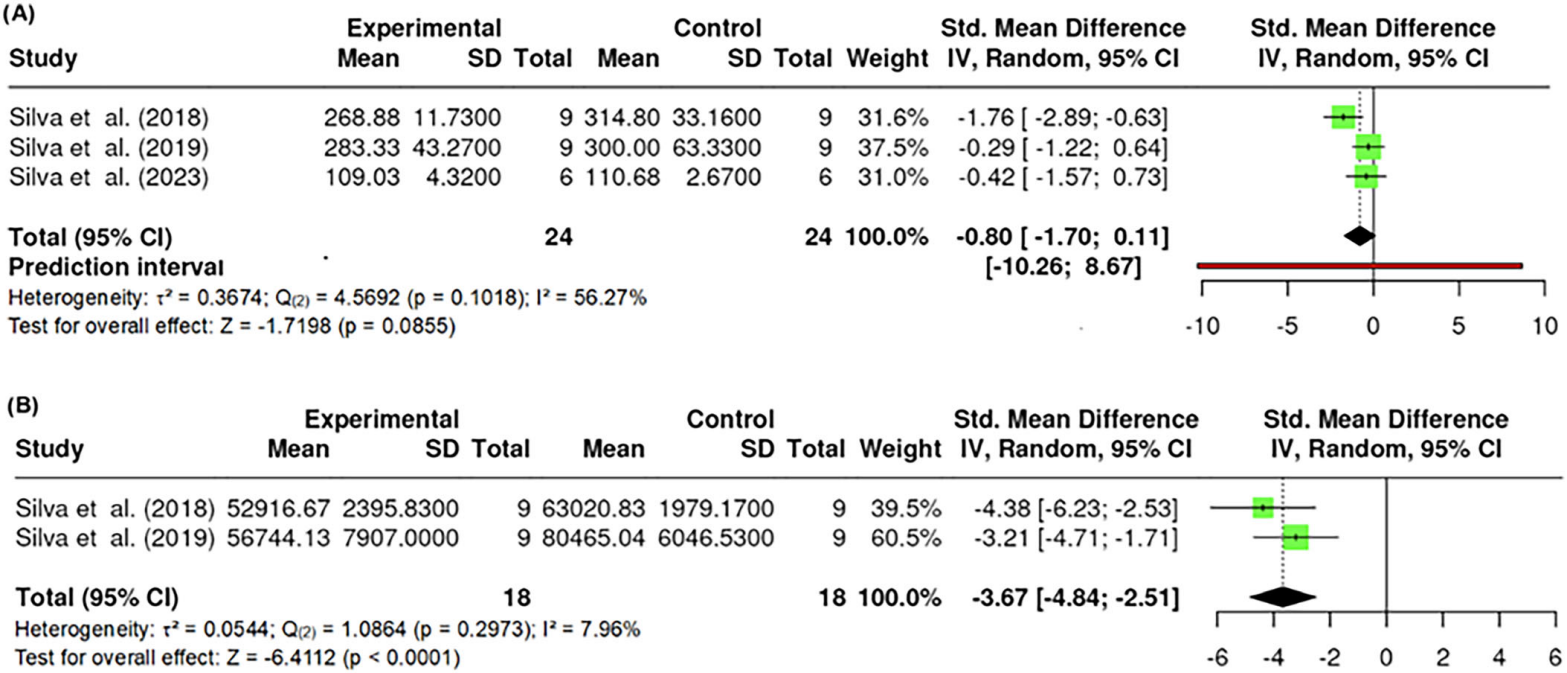
**Meta‐analysis using a random effects model of selected studies relating to glucose levels (A; p = 0.0855) and AUC (B; p < 0.0001) in mice models assessed in the studies included.** The plot shows the SMD and corresponding 95% CI for each study included in the meta‐analysis. The vertical dashed line represents the overall estimated effect size. Horizontal lines indicate the 95% CI for each study, with squares representing the individual study estimates, where the size of each square is proportional to the weight of the study in the analysis. The rhombus at the bottom represents the overall weighted effect size. The figure was generated using RevMan software.

The assessment of glucose levels included k = 3 studies, with estimated effect sizes ranging from 1.7172 to 0.1121. Moderate heterogeneity was observed (Q _(2)_ = 4.5692, p = 0.1018, τ^2^ = 0.3674, I^2^ = 56.27%). Neither Egger's test (p = 0.6935) nor Begg and Mazumdar's rank correlation test (p = 1.0000) showed evidence of publication bias or significant asymmetry. The overall estimate was not statistically significant (Z = −1.7198, p = 0.0855), indicating a lack of impact of PBM on glucose levels (Figure 19A).Two studies were included (k = 2) for the area under the curve (AUC) of glucose during the intraperitoneal glucose tolerance test (ipGTT). The estimated effect sizes ranged from −4.8213 to −2.5636, demonstrating low heterogeneity (Q _(2)_ = 1.0864, p = 0.2973, τ^2^ = 0.0544, I^2^ = 7.96%). The Begg and Mazumdar rank correlation test (p = 1.0000) indicated no evidence of publication bias or significant asymmetry. The overall estimate was statistically significant (Z = −6.4112, p < 0.0001), indicating a meaningful effect of PBM on AUC in these animal models (Figure 19B).

**FIGURE 20:**
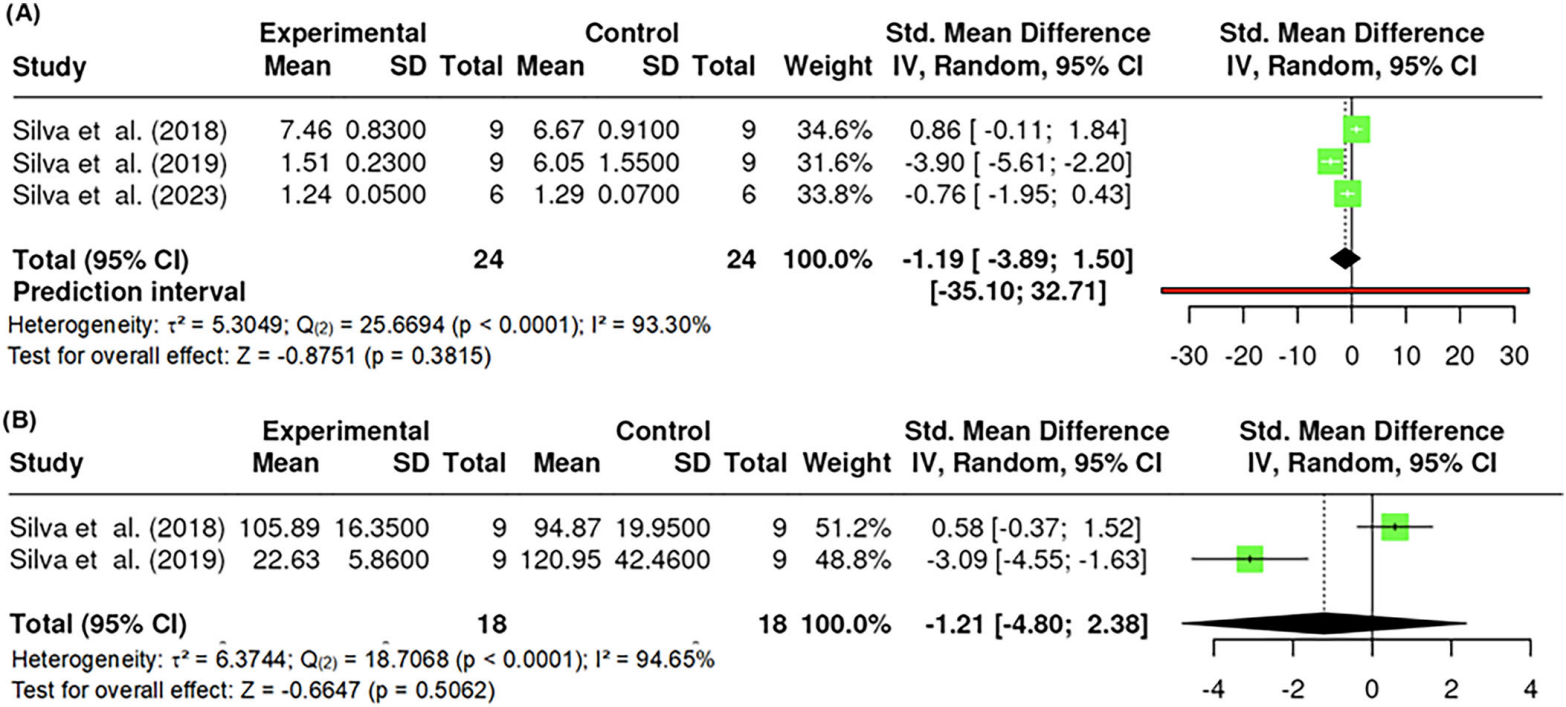
**Meta‐analysis using a random effects model of selected studies relating to insulin (A; p = 0.3815) and HOMA‐IR (B; p = 0.5062) in mice models assessed in the studies included.** The plot shows the SMD and corresponding 95% CI for each study included in the meta‐analysis. The vertical dashed line represents the overall estimated effect size. Horizontal lines indicate the 95% CI for each study, with squares representing the individual study estimates, where the size of each square is proportional to the study's weight in the analysis. The rhombus at the bottom represents the overall weighted effect size. The figure was generated using RevMan software.

The analysis of insulin levels involved k = 3 studies. Estimated effect sizes ranged from −3.9136 to 1.4975, with high heterogeneity observed (Q _(2)_ = 25.6694, p < 0.0001, τ^2^ = 5.3049, I^2^ = 93.30%). Egger's test (p = 0.0063) indicated significant evidence of publication bias, while Begg and Mazumdar's rank correlation test (p = 0.3333) showed no evidence of significant asymmetry. The overall estimate was not statistically significant (Z = −0.8751, p = 0.3815), suggesting no significant effect of PBM on insulin levels (Figure 20A).

Regarding HOMA‐IR, k = 2 studies were included in this analysis. The estimated effect sizes ranged from −4.8154 to 2.3763, with high heterogeneity (Q _(2)_ = 18.7068, p < 0.0001, τ^2^ = 6.3744, I^2^ = 94.65%). The Begg and Mazumdar rank correlation test (p = 1.0000) indicated no evidence of publication bias or significant asymmetry. The overall estimate was not statistically significant (Z = −0.6647, p = 0.5062), indicating no substantial effect of PBM on HOMA‐IR in these models (Figure 20B).

### 3.2 Method quality assessment

The quality of the included studies varied, with several showing concerns in different areas. Of the 17 clinical research studies assessed, five exhibited issues with the randomization process. For example, Da Silveira et al (2015)^38^ and Modena et al (2022)^33^ demonstrated a high risk of bias due to non‐random assignment, which may compromise the validity of their findings. Similarly, five studies, including Duarte et al (2015)^39^ and Nagy et al (2018),^34^ had concerns regarding the measurement of outcomes, suggesting potential inconsistencies in how results were evaluated.

In terms of missing outcome data, two studies such as Modena et al (2022)^33^ and Modena et al (2023),^13^ reported significant missing data, increasing the risk of bias. Moreover, the selection of reported results raised concerns in several studies, such as Croghan et al (2020)^30^ and Da Silveira et al (2018),^8^ where selective reporting could have influenced the findings.

On a positive note, several studies, including Mostafa et al (2016)^40^ and Hassan et al (2014),^29^ showed a low risk of bias across all domains, indicating higher methodological quality. However, the presence of possible limitations and the variability in risk across domains should be considered when interpreting the overall findings of these studies (see Table 2 for more details).

**TABLE 2:** Included clinical research studies rated against the Cochrane risk of bias tool.

Source	Randomization process	Deviations from the intended interventions	Missing outcome data	Measurement of the outcome	Selection of the reported results
Croghan et al (2016)^7^	**+**	**+**	**+**	**+**	**+**
Croghan et al. (2020)^30^	**+**	**+**	**+**	**+**	**!**
Da Silveira et al. (2015)^38^	**−**	**+**	**+**	**!**	**+**
Da Silveira et al. (2018)^8^	**−**	**+**	**+**	**!**	**!**
Duarte et al. (2015)^39^	**+**	**+**	**+**	**!**	**−**
Elkablawy et al. (2016)^14^	**−**	**+**	**+**	**!**	**+**
Elm et al. (2011)^44^	**−**	**+**	**+**	**!**	**+**
Elnaggar et al. (2020)^31^	**+**	**+**	**+**	**+**	**+**
Elsherbeni et al. (2018)^32^	**−**	**+**	**+**	**!**	**!**
Hassan et al. (2014)^29^	**+**	**+**	**+**	**+**	**+**
Modena et al. (2022)^33^	**−**	**+**	**−**	**−**	**!**
Modena et al. (2023)^13^	**−**	**+**	**−**	**−**	**!**
Mostafa et al. (2016)^40^	**+**	**+**	**+**	**+**	**+**
Nagy et al. (2018)^34^	**−**	**+**	**+**	**!**	**!**
Nagy et al. (2021)^35^	**+**	**+**	**+**	**+**	**+**
Roche et al. (2017)^36^	**+**	**+**	**+**	**+**	**+**
Said et al. (2016)^42^	**+**	**+**	**+**	**+**	**+**
Tseng et al. (2015)^37^	**+**	**+**	**!**	**−**	**!**

*Note*: + = Low risk of bias; − = High risk of bias;! = Some concerns.

## 4 DISCUSSION

Considering the widespread occurrence of obesity and the challenges associated with adherence and relapse following diverse treatments, it becomes imperative to explore potential synergies with supplementary non‐invasive interventions such as PBM. In this regard, the results presented in the current meta‐analysis, covering diverse anthropometrical, physiological, and psychological dimensions, underscore the importance of delving into the underlying mechanisms to enhance understanding of the observed outcomes. This comprehensive perspective aims not only to bolster the scientific evidence base in the field of PBM but also to pave the way for the development of more effective and personalized therapeutic strategies addressing a wide range of health conditions.

This review is focused on the application of PBM to the abdominal region due to the importance of this area in metabolic regulation and its close relationship with fat accumulation, which is a key factor in obesity and various metabolic disorders,^48^ making the abdomen a highly relevant area for studies evaluating therapeutic interventions such as PBM. Moreover, while PBM has been more studied in other regions of the organism such as the brain for treating neurological disorders,^49^ in dermatology for skin‐related conditions,^50^ or in muscle tissue,^51^ the systemic effects and specific mechanisms of PBM in the abdomen are still not fully understood. Therefore, our objective was to highlight the outcomes of abdominal PBM, specifically targeting obesity‐related alterations, including anthropometrical, physiological, and psychological variables.

Firstly, regarding the anthropometrical variables analyzed, the results show that PBM did not have a significant impact on various anthropometrical measurements, including weight, lean, and fat mass, as well as measurements of the distribution of body fat. Similar results were found in mouse models, where there were no significant differences in weight between groups. However, significant effects were observed for waist and hip circumferences in human models, and in the epididymal adipocyte area in mice models. These findings could indicate a specific influence of PBM on the regional distribution of body fat, which could have relevant implications for metabolic health. The significant changes observed in hip and waist circumference, contrasted with the lack of effect on overall body weight, suggest that PBM may have a more pronounced impact on the redistribution of body fat rather than on the total quantity of fat. Thus, the increase in hip circumference may indicate a redistribution of fat toward peripheral areas, which is considered metabolically favorable due to its association with a lower risk of metabolic and cardiovascular diseases.^52^ These findings suggest that PBM may encourage a fat distribution pattern that supports better metabolic health outcomes.

Studies by other authors^44^, ^53^, ^54^ have explored the effectiveness of PBM for body contouring with contradictory results. In Elm et al^44^ it was not shown to be effective in reducing the circumference of the treated areas or decreasing fat layer thickness when applying 635 nm (15 mW each diode). On the contrary, Jackson et al^54^ demonstrated that PBM of the appropriate wavelength (635 nm 2.5 mW each diode) applied three times per week for 2 weeks can significantly reduce the circumference at specifically targeted tissue sites due to reduction in the adipose layer. As suggested mechanisms, Jackson et al^54^ showed that PBM can lead to the upregulation of reactive oxygen species (ROS), which impacts the cellular redox state and activates redox‐sensitive transcription factors, such as NF‐kB and AP‐1,^55^, ^56^ and phospholipase A2.^57^ This shift toward an oxidized state promotes gene expression through specific signaling pathways.^58^ The activation of these transcription factors may influence membrane‐related proteins, potentially altering the permeability of adipocytes.^54^

Another suggested mechanism of PBM on lipid metabolism is its effect on lipid peroxidation, the oxidative breakdown of membrane‐bound cholesterol, which negatively impacts membrane structure and function.^59^ Research has shown that exposure to low‐energy laser irradiation can trigger the upregulation of secondary free radical reactions, leading to increased lipid peroxidation.^60^, ^61^

The third suggested mechanism by Neira et al^62^ and Caruso et al^53^ proposed that PBM treatment did not kill fat cells but led to the increased presence of pores in cell membranes. When human fat cells were exposed to PBM treatment without serum, no triglycerides were released, but with normal or heat‐inactivated serum, triglyceride levels in the media increased. This suggests that the presence of serum simulates an in vivo environment, allowing PBM to promote fat loss. As a result, there may be a reduction in adipocyte size, leading to decreased body fat, particularly in areas like the waist, thereby enhancing body contouring.^53^

Fat mobilized by PBM treatment likely enters the bloodstream via lymphatics, in a similar manner to how dietary fat is absorbed. If body weight remains stable, the mobilized fat is either burned for energy or redistributed to typical fat storage areas. It is important to note that PBM treatments do not alter the body's natural fat distribution patterns, so without ongoing treatments, fat will redistribute normally.^53^

The influence of PBM on physiological variables appears to be variable, with significant effects on triglycerides and insulin levels and no effects on the levels of fasting glucose, lipoproteins, and total cholesterol. These results could suggest a potential role of PBM in the regulation of glycemic homeostasis, which may be related to its ability to modulate cell signaling, specifically through pathways such as the insulin receptor pathway, and could indicate a specific impact on insulin sensitivity, as reflected in the HOMA‐IR index. This finding is especially interesting in the context of preventing insulin resistance and associated metabolic diseases. Studies have suggested that PBM may improve insulin sensitivity and promote glucose uptake through activation of the insulin receptor pathway^63^ and phosphatase and tensin homolog (PTEN)/protein kinase B (AKT) signaling. Irradiation with light at 635 nm wavelengths has been observed to enhance insulin receptor phosphorylation and increase insulin receptor tyrosine kinase activity, leading to increased translocation of GLUT4 glucose transporters to the membrane and increased glucose uptake in peripheral tissues, such as skeletal muscle and adipose tissue.^45^ Specifically, PBM is known to raise CCO activity, which in turn raises the production of ROS and ATP. It has been shown that PBM triggers ROS‐induced signaling through protein kinase B (AKT)/phosphatase and tensin homolog (PTEN) which facilitates the translocation of the glucose transporter GLUT4 to the cell membrane. Additionally, it has been proposed that PBM‐induced PTEN/AKT signaling also stimulates glycogen synthase activation, speeding up glycogen synthesis in skeletal muscle. This action contributes to the maintenance of glycemic balance and lipid metabolism, suggesting a possible beneficial effect of PBM on insulin sensitivity and the regulation of glucose metabolism.^64^

Moreover, our results may highlight a specific effect of PBM on lipid metabolism, as changes in triglyceride levels were observed in both animal and human models. Sattarinezhad et al (2021)^65^ observed that lower triglyceride levels are related to higher adiponectin levels. Several mechanisms may be involved in this effect. Adiponectin might increase serum level and activity of the lipoprotein lipase enzyme^66^, ^67^ or might induce VLDL receptor expression and consequently increase VLDL catabolism.^68^ However, our meta‐analysis, did not show significant changes in LDL‐cholesterol which is in line with the lack of correlation between serum levels of adiponectin and LDL‐cholesterol observed in other studies.^65^, ^69^, ^70^, ^71^, ^72^ It is important to note that the impact of PBM on this correlation could not be further explored in this meta‐analysis as most of the studies did not address it.

Finally, regarding the psychological variables, no significant effects of PBM were observed on perceived quality of life or body image. The lack of effect on these measurements could suggest that while PBM may have physical benefits, its impact on subjective perception and psychological well‐being may be limited. However, it is important to note that very few studies included an assessment of psychological variables^7^, ^30^ and they employed wavelengths under 600 nm (such as the 532 nm green wavelength).^7^, ^30^ This phenomenon of non‐significant results is explained in these studies by unrealistic expectations of greater weight loss after PBM among participants. Indeed, Croghan et al^7^ suggest the inclusion of an initial intake discussion to set realistic expectations in order to maximize the impact of the study and improve adherence to the program. Therefore, it is necessary for all studies to evaluate the effect of PBM on neuropsychological variables and it is essential to investigate the potential outcomes with other wavelengths, including red and near‐infrared, which have shown positive results on various variables.

In this respect, dosimetry plays a key role in fat reduction treatments, as different doses and wavelengths trigger specific cellular responses, and several studies emphasize the importance of tailoring these parameters for optimal results.^73^, ^74^, ^75^ For instance, Moon (2022)^73^ and Montazeri (2017)^75^ both explored the use of dosimetry in fat reduction and revealed interesting results. The study of Moon (2022)^73^ used a 1060 nm diode laser with 635 nm, resulting in fat reductions of up to 26.4%, while the study of Montazeri (2017)^75^ employed 630 nm, 808 nm, and 450 nm wavelengths. Each wavelength contributed uniquely: red light‐induced biochemical changes in adipocytes, infrared improved tissue penetration, and blue light enhanced nitric oxide release, improving tissue perfusion. These findings underline how different wavelengths, used in combination, can synergistically boost fat reduction at the cellular level, with dose optimization being crucial for success.

Our results highlight the need to consider diversity in the response to PBM across different variables and the importance of additional research to better understand potential underlying mechanisms and moderating factors. For example, high variability was found in the methods used, including LipoLaser,^14^, ^34^, ^35^, ^44^ cryolipolysis,^40^ ultrasonic cavitation,^14^ and acupuncture points.^37^, ^76^ In this context, the differential impact of wavelengths on BMI is worth mentioning. A significant effect on BMI reduction was observed when wavelengths around 600 nm were applied (635 and 660 nm), whereas wavelengths below 600 nm or around 800 nm did not show significant effects. However, it is important to note the high heterogeneity among studies using wavelengths in the 600 nm range, which could be explained by other relevant variables in PBM application. Regarding the participants in the included studies in the 600 nm range, we found a large heterogeneity between the articles, with men and women subjects aged 65–75 years,^35^ boys and girls participants aged 13–16 years,^31^ and a population exclusively of women aged 30–40 years.^14^ PBM may have a different effect depending on age group and gender, as has been found in other previous studies. Gender, in particular, appears to be a crucial variable in individual responses to PBM therapy, influencing outcomes and even mitochondrial gene expression.^77^ Additionally, some studies have reported differing CCO and hemodynamic activity and neuroinflammation response after PBM in older populations compared to younger groups.^78^, ^79^ Differences in BMI, therefore, could be due to the fact that in older adults, a slower metabolism^80^ may make PBM less effective in reducing body fat and lowering BMI. It is also important to highlight that the studies using wavelengths around 600 nm employed various commercial light sources, with differing PBM parameters and time durations. This issue is important, as different light sources may vary in power, coherence, irradiation intensity, and treatment area size, which may result in a different impact on obesity patients. Even small differences in wavelength can influence the depth of light penetration and cellular effects on adipose tissue, contributing to variability in outcomes.^81^ Indeed, this wavelength variability could not be studied with sub‐analyses for body image or body image satisfaction or with the other physiological variables because most of them used a wavelength of 800 nm. Moreover, other parameters such as the energy density, output power, application time, and the number of repetitions of the light application could impact therapeutic success. However, despite the variability in wavelengths, the consistent and significant results in variables like waist and hip circumference, as well as triglyceride and insulin levels, suggest a robust response to PBM in these specific measures.

Finally, the double‐blind design of this study represents a critical methodological strength, as it eliminates expectation bias by ensuring that neither participants nor investigators were aware of the treatment allocation. This approach minimizes the risk of subjective influences on the outcomes, thereby improving the internal validity and robustness of the findings.

## 5 CONCLUSION

Transabdominal applied PBM demonstrates discernible effects on anthropometrical, psychological, and physiological parameters in individuals with obesity. Regarding anthropometrical metrics, PBM exhibits potential efficacy in influencing waist and hip circumference, suggesting a possible impact on body shape and contouring. However, no statistically significant differences were observed in weight, BMI, waist‐to‐hip ratio, neck circumference, skin fold, lean mass, fat mass, or visceral mass. In terms of psychological variables, there were no discernible differences in LASA and body image. Lastly, physiological measures revealed a significant impact on insulin levels and triglycerides. Conversely, no changes were noted in HDL, LDL, cholesterol, HOMA‐IR, leptin, and platelet levels. This study emphasizes the necessity of considering diverse wavelengths in PBM research, especially in the context of obesity treatment. It also underscores the imperative for further investigation to comprehensively understand the mechanisms and applications of PBM. This holistic perspective provides valuable insights into the fundamental mechanisms of PBM in individuals with obesity and its overarching impact on health and well‐being.

## CONFLICT OF INTEREST

Authors declare no conflicts of interest to disclose.
